# MiR-34 inhibits polycomb repressive complex 2 to modulate chaperone expression and promote healthy brain aging

**DOI:** 10.1038/s41467-018-06592-5

**Published:** 2018-10-10

**Authors:** Jason R. Kennerdell, Nan Liu, Nancy M. Bonini

**Affiliations:** 1 Department of Biology, University of Pennsylvania, Philadelphia, PA 19104 USA; 2 Present Address: Interdisciplinary Research Center on Biology and Chemistry, Shanghai Institute of Organic Chemistry, Chinese Academy of Sciences, Shanghai, 201210 China

## Abstract

Aging is a prominent risk factor for neurodegenerative disease. Defining gene expression mechanisms affecting healthy brain aging should lead to insight into genes that modulate susceptibility to disease. To define such mechanisms, we have pursued analysis of *miR-34* mutants in *Drosophila*. The *miR-34* mutant brain displays a gene expression profile of accelerated aging, and *miR-34* upregulation is a potent suppressor of polyglutamine-induced neurodegeneration. We demonstrate that *Pcl* and *Su(z)12*, two components of polycomb repressive complex 2, (PRC2), are targets of *miR-34*, with implications for age-associated processes. Because PRC2 confers the repressive H3K27me3 mark, we hypothesize that *miR-34* modulates PRC2 activity to relieve silencing of genes promoting healthful aging. Gene expression profiling of the brains of hypomorphic mutants in *Enhancer of zeste* (*E(z)*), the enzymatic methyltransferase component of PRC2, revealed a younger brain transcriptome profile and identified the small heat shock proteins as key genes reduced in expression with age.

## Abstract (web summary)

miR-34 is known to regulate age-related gene expression in the *Drosophila* brain, and miR-34 overexpression can attenuate neurodegeneration induced by polyQ-expanded proteins. Here, Kennerdell and colleagues show that miR-34 confers longevity and neuroprotection via an epigenetic regulator Polycomb Repressive Complex 2 and molecular chaperone expression.

## Introduction

Aging is an important major risk factor for developing neurodegenerative disease. However, molecular pathways that confer susceptibility to aged individuals are still being explored. Several genetic manipulations can produce long-lived animals: for example, *daf-2* mutations extend lifespan in *C. elegans*^1^, as does upregulation of a sirtuin in both *C. elegans* and *S. cerevisiae*^2,3^. Non-genetic interventions also extend lifespan, including caloric restriction and ablation of the reproductive tissue in *C. elegans*^4,5^. Better understanding of mechanisms that confer a healthy brain could lead to insight for treatments for age-associated brain diseases.

The microRNA *miR-34* is a post-transcriptional regulatory molecule that impacts age-associated outcomes in *Drosophila*. *MiR-34* becomes upregulated in the fly brain in an age-associated manner and is functionally required for healthy brain aging^6^. The mutants undergo brain degeneration and die prematurely; transcriptional profiling demonstrates an accelerated age-associated profile of transcriptional changes^6^. As such, *miR-34* mutant animals display normal age-associated processes, but at an accelerated pace. Consistent with a role in healthy brain aging, upregulation of *miR-34* in the fly extends median lifespan and potently mitigates degeneration in a model of polyglutamine disease. Elucidating the targets of *miR-34* that help confer these functions would lead to insight into mechanisms of healthy brain aging.

MicroRNAs affect protein abundance by binding select target mRNAs containing sequence complementarity within their 3′UTR. This results in decreased translation or degradation of the mRNA, leading to a downregulation of the protein. Several activities of *miR-34* can be attributed to regulation of *Eip74EF*, a component of steroid hormone signaling pathways^6^. However, *Eip74EF* does not affect polyglutamine toxicity, suggesting that other targets of *miR-34* are involved in regulating age-associated susceptibility to brain disease. Identification of additional targets of *miR-34* should provide additional insight into aging of the brain, and define mechanisms that can delay or prevent age-associated diseases. A number of observations have implicated *miR-34* in the response to stress: *miR-34* mutants are sensitive to stress in flies and in *C. elegans*^6,7^. In flies, *miR-34* brain tissue also accumulates inclusions of misfolded protein^6^, and upregulation of *miR-34* activates innate immunity pathways and promotes survival from infection^8^. These data suggest that *miR-34* function should impact stress-associated genes important for maintaining health of the brain.

The importance of epigenetic modulation of the genome has been well established for developmental events; however, the role and impact on aging and age-associated diseases is only recently gaining attention^9^. Histone modification pathways affect lifespan in yeast^10,11^, and mutations in components of the H3K4 trimethylation complex extend lifespan in *C. elegans*^12^. Mutation in components of polycomb repressive complex 2 (PRC2), which confers the H3K27me3 mark associated with decreased gene expression, extends lifespan and promotes resilience to stressors^13,14^. PRC2 is elevated in a mouse model of ataxia-telangiectasia, and inhibiting PRC2 activity suppresses neurodegeneration in this model^15^. Despite these findings, little is known mechanistically regarding epigenetic modulation in the brain with age, yet such modulations could have a vast impact on healthy aging, stress resistance, and disease susceptibility.

In pursuing additional targets of *miR-34* that may contribute to healthy brain aging, we reveal that *miR-34* regulates two components of the PRC2 complex—Su(z)12 and Pcl—to convey resistance to polyglutamine toxicity. By study of potential epigenetically modulated genes in the brain with age normally and upon PRC2 reduction, we reveal that small heat shock proteins (sHSPs) may be critical players in healthy brain aging. Protein chaperones, including the sHSPs, have been shown to be particularly important in protection from neurodegeneration, stress, and age-associated processes in both flies and *C. elegans*^16–23^. Detailed transcriptomic analysis further reveals that reduction of PRC2 function in the fly brain confers a chronologically “younger” transcriptomic profile, opposite to that of the *miR-34* mutant^6^, highlighting that approaches to modulate the epigenome may impact healthy aging of the brain.

## Results

### *MiR-34* regulates the *Pcl* and *Su(z)12* 3′UTRs

**Fig. 1:**
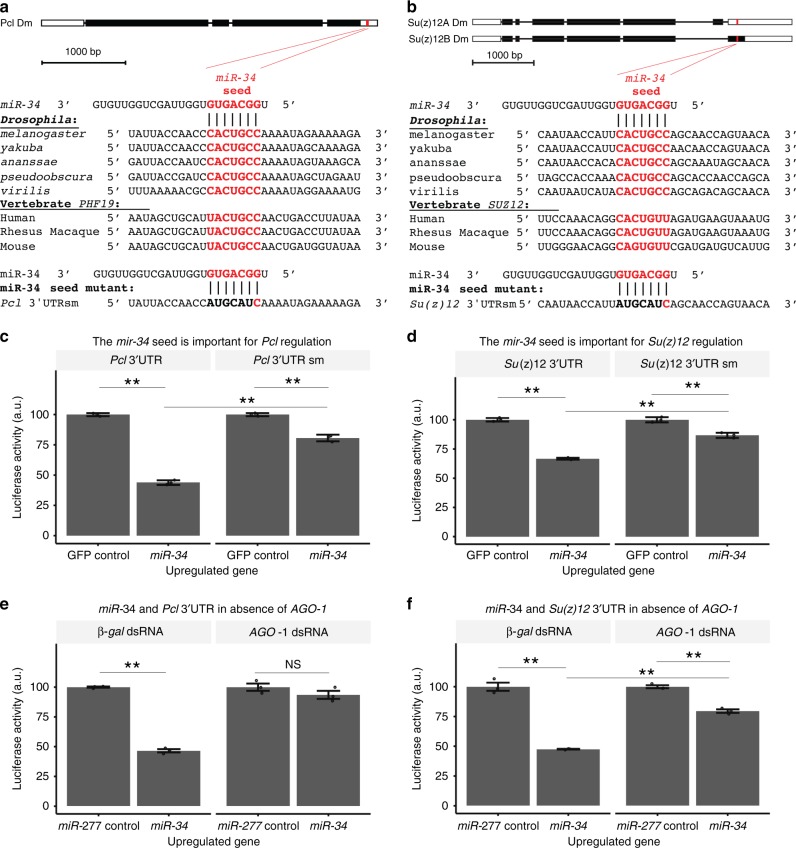
miR-34 targets 3′UTRs of Su(z)12 and Pcl. **a**, **b** *Pcl* and *Su(z)12* are predicted targets of *miR-34*. The seed sequence of *miR-34* has complementarity with the (**a**) *Pcl* 3′UTR and the (**b**) *Su(z)12* 3′UTR. The seed sequence is conserved in various homologs. Bottom, an engineered mutation in the seed sequence was also generated, red letters indicate base-pairing retained in the seed mutation, those with disrupted base-pairing are in black. **c**, **d** Luciferase assays confirm that *miR-34* targets *Pcl* and *Su(z)12*. A reporter construct containing the 3′UTR of (**c**) *Pcl* or (**d**) *Su(z)12* fused to Renilla luciferase was expressed in *Drosophila* SL2 cells and tested for a response to *miR-34*. Upon *miR-34* upregulation, luciferase activity decreases in reporters containing the (**c**, left) *Pcl* 3′UTR and the (**d**, left) *Su(z)12* 3′UTR. Regulation of the *Pcl* 3′UTR by *miR-34* was attenuated by a seed sequence mutation (**c**, right). Regulation of the *Su(z)12* 3′UTR by *miR-34* was also attenuated by the seed sequence mutation (**d**, right). Two-way ANOVA indicated a significant interaction term for the *Pcl* 3′UTR (F_1,8_ = 8.5, *p* < 0.02), and *Su(z)12* 3′UTR (F_1,8_ = 17.5, *p* < 0.01). **p < 0.01, two-way ANOVA with Tukey post-test. Mean ± SEM, *n* = 3 wells. **e**, **f** The RNAi induced silencing complex (RISC) is required for regulation of the 3′UTR of *Pcl* and *Su(z)12* by *miR-34*. The 3′UTR reporter constructs were expressed in DL1 cells treated with dsRNA to *argonaute-1* (an essential component of RISC) or a control gene (*β-gal*). The cells were tested for a response to upregulation of *miR-34* or a control microRNA (*miR-277*). In cells treated with *β-gal* dsRNA, upregulation of *miR-34* resulted in decreased reporter expression. In cells treated with *AGO1* dsRNA, the effect of miR-34 on reporter expression was eliminated (**e**, for *Pcl* 3′UTR reporter two-way ANOVA indicated a significant interaction term, F_1,8_ = 107, *p* < 0.01) or attenuated (**f** for *Su(z)12* 3′UTR reporter two-way ANOVA indicated a significant interaction term, F_1,8_ = 73, *p* < 0.01). **p < 0.01, Two-way ANOVA, Tukey post-test. Mean ± SEM, *n* = 3 wells

To define targets of *miR-34* that might be important to brain aging, we scanned the lists of potential target genes using multiple microRNA prediction algorithms (Supplementary Table 1). We noted that two components of the same complex, PRC2, are targets: *Polycomblike* (*Pcl*), a peripheral component of PRC2^24^, is a predicted target in *Drosophila* and vertebrates; and a gene encoding a protein within the core PRC2 complex, *Su(z)12*. Polycomb group proteins function together as members of several repressive complexes called PRC1, PRC2, and PRC2-Pcl, of which the protein subunits can vary^25^. Methylation of lysine 27 of histone H3 by the enzymatic methyltransferase component of PRC2, Enhancer of zeste (E(z)), recruits PRC1 to select loci typically resulting in transcriptional silencing, although it may also result in increased transcription depending on the distribution of the epigenetic mark^26^. The *miR-34* binding site appears conserved in all *Drosophilae* examined, and is also conserved in one of the three human orthologues of *Pcl*, *PHF19* (Fig. 1a). To determine whether *miR-34* can regulate *Pcl* through this site, we used a luciferase cell culture reporter assay. A *Renilla* luciferase reporter utilizing the 3′UTR of *Pcl* was used to assess the sensitivity of the 3′UTR to *miR-34* in SL2 cells. Upregulation of *miR-34* was achieved with an expression construct driven by an inducible metallothionein promoter. Upregulation of *miR-34* resulted in a 56% decrease in reporter expression with the 3′UTR of *Pcl* when compared to a control inducing expression of GFP (Fig. 1c, left; also Supplementary Figure 1). To determine whether the decrease in reporter activity was dependent upon the *miR-3*4 seed region, we generated a seed mutant that disrupted strand complementarity (Fig. 1a, bottom). The seed mutation reduced the ability of *miR-34* to regulate the *Pcl* 3′UTR (Fig. 1c, right). These data indicate that the *Pcl* 3′UTR can be regulated by *miR-34*, at least in part through the seed region defined.

In parallel, we examined *miR-34* regulation of *Su(z)12*, the rate limiting component of PRC2^27^. Previous work investigating the reliability of a microRNA prediction algorithm demonstrated that upregulation of *miR-34* decreases translation of a reporter bearing the 3′UTR of *Su(z)12*^28^. *MiR-34* regulation is not predicted in mammalian *SUZ12* by common algorithms; however, RNAhybrid^29^ showed a potential site in the 3′UTR of vertebrate *SUZ12* (Fig. 1b). Targeting of *SUZ12* through this cryptic site would require wobble base pairing which is tolerated in some miRNA/target pairings^30^. We confirmed that *miR-34* regulates the 3′UTR of fly *Su(z)12* and determined that this regulation was dependent on the *miR-34* seed sequence. Upregulation of *miR-34* resulted in a 33% decrease in the reporter utilizing the 3′UTR of *Su(z)12* (Fig. 1d, left; also Supplementary Figure 1), consistent with the previous findings. A *Su(z)12* reporter with a mutated 3′UTR that disrupted strand complementarity between *miR-34* and the 3′UTR of *Su(z)12* (Fig. 1b, bottom) yielded an attenuated response. These data suggest that *miR-34* regulation requires, at least in part, the seed sequence present in the 3′UTR of *Su(z)12*.

If this regulation of the *Pcl* and *Su(z)12* 3′UTR reporter constructs occurs upon upregulation of the mature *miR-34* miRNA, then we would expect interfering with the miRNA maturation pathway should reduce the effect of *miR-34* induction on reporter expression. We shifted our experimental model to DL1 cells, which gave a more robust RNAi response compared to SL2 cells. Treatment of the cells with dsRNA against *argonaute-1 (AGO1)*, a gene essential for miRNA maturation, or against *β-galactosidase* as a control, resulted in loss of regulation by *miR-34* compared to a control miRNA *miR-277*, for the *Pcl* 3′UTR (Fig. 1e). MiRNA maturation was also required for regulation of the *Su(z)12* reporter by *miR-34*: in cells treated with *β-galactosidase* dsRNA control, upregulation of *miR-34* decreased the *Su(z)12* reporter activity by 52% (Fig. 1f, left), but when cells were treated with dsRNA against *AGO1*, regulation by *miR-34* was diminished to 19% (Fig. 1f, right). Taken together, these data indicate that *miR-34* has activity to regulate the levels of 3′UTR reporter constructs for the PRC2 complex components *Pcl* and *Su(z)12* in cell culture assays.

### *MiR-34* regulates Pcl and Su(z)12 levels in the aging brain

**Fig. 2:**
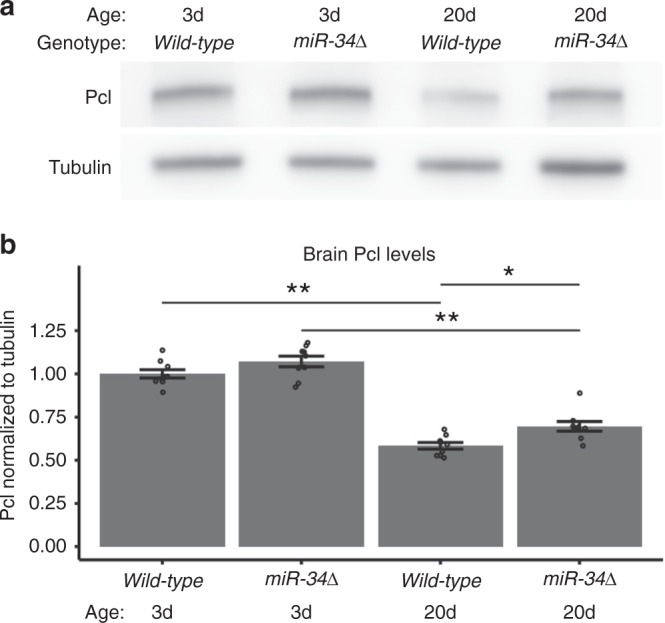
*Pcl* is a target of *miR-34* in the brain. **a**, **b** Pcl protein levels are deregulated in *miR-34* mutants. **a** Western immunoblots. **b** Quantitation of immunoblots. Pcl levels decrease with age in both wild-type and *miR-34* mutants. Pcl levels were higher than wild-type control at 20d. Protein samples from dissected brains. Western immunoblot, with tubulin as the loading control. Pcl protein levels are normalized to tubulin. Mean ± SEM, *n* = 9 biological replicates. Significant main effects were observed (genotype: F_1,32_ = 12.7, *p* < 0.01 age: F_1,32_ = 232, *p* < 0.001). (*p < 0.05, **p < 0.01, two-way ANOVA with Tukey post-test)

Given this regulation in cells, we extended the findings to the brain in vivo. *MiR-34* is expressed in the *Drosophila* brain in an age-dependent manner, with low expression in young adult animals and increased expression with age^6^. We confirmed specificity of an anti-Pcl antibody by verifying that an immunoband present in brain tissue was depleted upon hypomorphic mutation or knockdown of *Pcl* (Supplementary Figure 2). Using this antibody, we examined the levels of *Pcl* protein in young (3d) and older (20d) fly brain tissue. As wild-type animals age, the level of *Pcl* protein in the brain decreased dramatically, dropping by 43% (Fig. 2a, b). This reduction was attenuated in 20d *miR-34* mutants such that it was 22% higher than in wild-type age-matched controls, consistent with regulation, albeit modest, of Pcl levels by *miR-34* at advanced age when *miR-34* expression levels are normally high (also Supplementary Figure 2). These data are consistent with some degree of regulation of the levels of Pcl in vivo with age by *miR-34*.

**Fig. 3:**
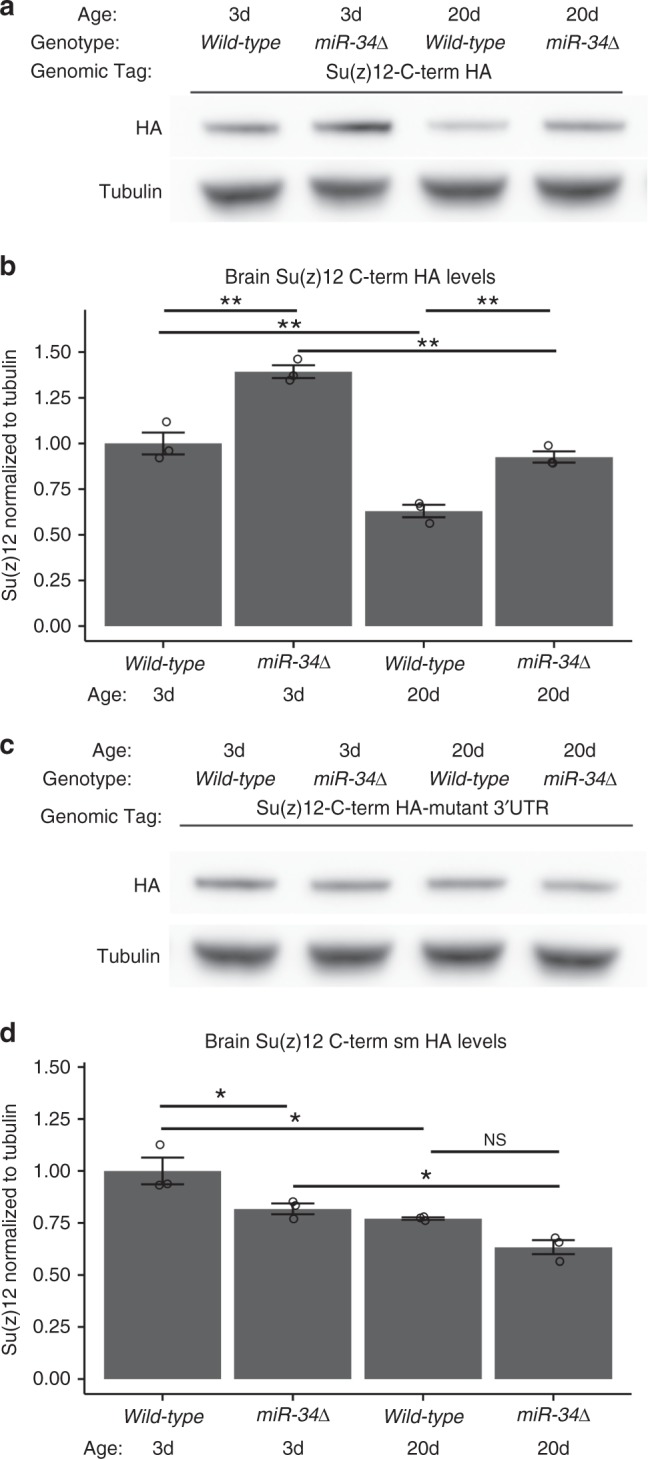
Su(z)12 is a target of *miR-34* in the brain. **a**, **b** Su(z)12 protein levels are deregulated in *miR-34* mutants. Su(z)12 protein was detected using a transgene containing the *Su(z)12* genomic region tagged with the HA epitope on the C-terminus of the predicted protein (Su(z)12-C-term HA). Su(z)12 levels decreased with age in wild-type, but remained elevated in *miR-34* mutants. Protein samples from dissected brains. Su(z)12-C-term HA protein levels were normalized to tubulin loading control. Mean ± SEM, *n* = 3 biological replicates. Significant main effects were observed (genotype: F_1,8_ = 68, *p* < 0.001 age: F_1,8_ = 101, *p* < 0.001). (*p < 0.05, **p < 0.01, two-way ANOVA with Tukey post-test).**c**, **d** The regulation of Su(z)12 protein levels by *miR-34* is dependent upon the *miR-34* seed sequence in the 3′UTR of the *Su(z)12* transcript. The Su(z)12-C-term HA-mutant 3′UTR transgene contains a mutation in the *miR-34* seed sequence that relieves the transcript from regulation by *miR-34* (see Fig. 1d). Su(z)12-C-term HA-mutant 3′UTR protein levels still decrease with age, but are no longer increased in *miR-34* mutants. The difference between wild-type at 20d and *miR-34* mutant at 20d is not significant. Protein samples from dissected brains. Quantification of Su(z)12-C-term HA-mutant 3′UTR protein levels using Western immunoblotting, normalized to tubulin loading control. Mean ± SEM, *n* = 3 biological replicates. Significant main effects were observed (genotype: F_1,8_ = 17, *p* < 0.01 age: F_1,8_ = 28.5, *p* < 0.001). (*p < 0.05, two-way ANOVA with Tukey post-test)

To determine whether *Su(z)12* was also an in vivo target of *miR-34*, we constructed a genomic transgene designed to express the *Su(z)12* protein tagged by a C-terminal HA epitope. To assess *Su(z)12* protein levels within its normal genomic context we included promoter, introns, and UTRs in the transgene. As with the Pcl protein, Su(z)12 protein levels normally decreased with age, becoming reduced by 37% from 3d to 20d in the wild-type brain (Fig. 3a, b; Supplementary Figure 3). In *miR-34* mutant tissue, the levels of HA-tagged Su(z)12 protein became elevated compared to age-matched controls, by 39% at 3d and 47% at 20d, indicating that *Su(z)12* is also an in vivo target of *miR-34* activity.

Having shown that Su(z)12 protein levels are higher in *miR-34* mutants, we determined whether this regulation was dependent on the *miR-34* seed sequence within the 3′UTR of the *Su(z)12* transcript. A mutation was introduced into the transgene to disrupt the base-pairing of *miR-34* in the 3′UTR (Fig. 1b, bottom) and transgenic animals generated. The mutated transgene was then crossed into *miR-34* mutants. This construct also showed decreased protein with age, by 23% in both wild-type and *miR-34* from 3d to 20d. However, the mutated transgene was no longer elevated in *miR-34*, and was mildly decreased (Fig. 3c, d; Supplementary Figure 3). These data indicate that the *miR-34* seed sequence in the 3′UTR of *Su(z)12* is important for proper regulation of Su(z)12 protein levels with age. Taken together, these findings suggest that *miR-34* targets the *Pcl* and *Su(z)12* transcripts, to help reduce the levels of these PRC2 complex proteins with age.

### Brain histone H3K27me3 levels are elevated in *miR-34* mutants

**Fig. 4:**
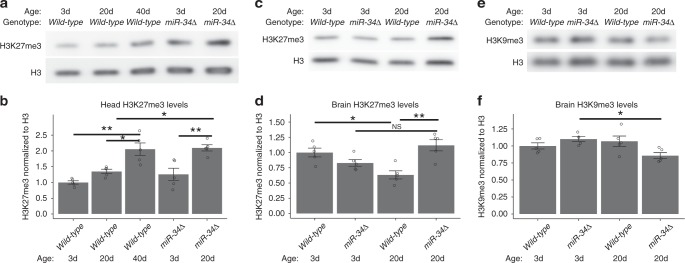
H3K27me3 levels with age and in *miR-34* mutants. **a**, **b** H3K27me3 levels with age in heads. Total histone was purified from *Drosophila* heads at 3d, 20d, and 40d. H3K27me3 gradually increased with age, and increased faster in *miR-34* mutants. Histone H3 is a control for total H3 levels. Quantification of relative levels of H3K27me3 was normalized to the total histone H3. Mean ± SEM, *n* = 5 biological replicates. Significant main effects were observed (genotype: F_1,20_ = 13.5, *p* < .01, age: F_2,20_ = 18, *p* < .001). (*p < 0.05, **p < 0.01 two-way ANOVA, with Tukey post-test).**c**, **d** H3K27me3 levels are increased in the brain of aged *miR-34* mutants. Protein samples were derived from dissected brains. H3K27me3 protein levels are normalized to total Histone H3 levels. Mean ± SEM, *n* = 5 biological replicates. Significant interaction and main effect were observed (genotype: F_1_, _16_ = 4.7, *p* < .05, age: F_1, 16_ = 0.28, ns, interaction: F_1, 16_ = 2.06, *p* < .001. (*p < 0.05, **p < 0.01 two-way ANOVA with Tukey post-test). **e**, **f** H3K9me3 levels are affected in *miR-34* mutants. Protein samples from dissected brains. H3K9me3 levels were normalized to total histone H3. Mean ± SEM, *n* = 5 biological replicates. A significant interaction was observed (interaction: F_1 16_ = 8.76, *p* < 0.01, genotype: F_1,16_ = 1.1, *p* > .05, age: F_1,16_ = .2.7, *p* > .05). (*p < 0.05, two-way ANOVA, Tukey post-test)

A key function of the PRC2 complex is to silence gene expression by methylating proximal histones with H3K27me3^31^. Thus, the increases in Su(z)12 and Pcl protein that we observed in *miR-34* mutants could result in downstream changes in H3K27me3. To examine this, we purified histones from young (3d) and aged (20d, 40d) *Drosophila* heads of wild-type and *miR-34* mutant animals, and used Western immunoblotting to assay total histone H3 and H3K27me3 modification levels. In wild-type animals, the levels of H3K27me3 rose steadily with age, increasing by 105% at 40d (Fig. 4a, b). In *miR-34* mutant animals, H3K27me3 also increased with age, but the increase was accelerated (67% from 3d to 20d for *miR-34*, compared to the wild-type trend of 35% from 3d to 20d). *MiR-34* mutant animals at 20d had H3K27me3 levels comparable to those of wild-type animals at 40d, and these levels were more than twice the levels of 3d wild-type animals (Fig. 4a, b).

We refined these observations by limiting the analysis to brain tissue. The brain is enriched for *miR-34* compared to heads^6^. We carefully dissected brains from the head capsule to assess levels in central brain plus outer optic regions, lacking the eyes and lamina. Interestingly, when examined in this manner, the levels of H3K27me3 in wild-type animals did not increase with age, but rather decreased (Fig. 4c, d). However, the increase of H3K27me3 in mutant compared to wild-type brains was still observed, with mutants displaying a 76% increase over age-matched wild-type brains. These data indicate that the increase in H3K27me3 observed in heads with age may be largely due to changes outside the central brain region dissected. We also addressed the specificity of histone modulation with age by assessing an additional histone silencing mark, H3K9me3, which, interestingly, was modestly affected by mutation of *miR-34* (Fig. 4e, f).

Taken together, these data suggest that *Pcl* and *Su(z)12* are in vivo targets of *miR-34*, and that the H3K27me3 activity of the PRC2 complex with which these proteins are associated is modulated with age in the brain. H3K27me3 levels are also higher in *miR-34* mutants with age than in wild-type tissue. These findings raised the possibility that modulation of PRC2 and H3K27me3 may be a molecular mechanism that impacts susceptibility of the brain to degenerative disease with age, given that *miR-34* mutants undergo brain degeneration and display an accelerated aging profile, and that upregulation of *miR-34* robustly prevents neurodegeneration^6^. Therefore, *miR-34* levels may increase with age to help minimize age-related elevations in H3K27me3 and negative impacts of this histone modification on pathways associated with neurodegeneration.

### *Pcl* and *Su(z)12* mutations mitigate polyglutamine toxicity

**Fig. 5:**
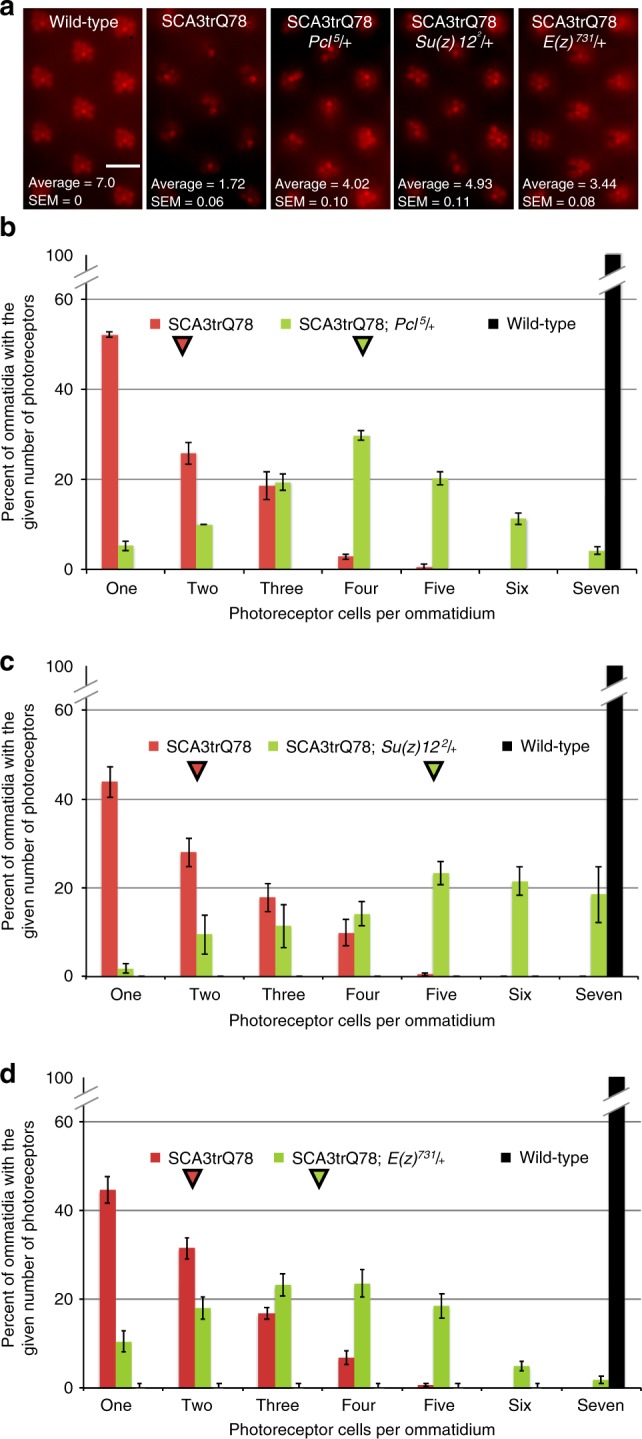
Neural degeneration due to pathogenic ATXN3 is suppressed by PRC2 mutations. **a** The fly compound eye is comprised of individual optical units called ommatidia, which are arranged in a hexagonal array. Within each ommatidium are eight photoreceptors (PR), of which seven are visible by this assay. Each panel shows eleven ommatidial units; flies are 21d old. Whereas wild-type has seven PRs in each ommatidial unit, expression of SCA3trQ78 results in PR loss. Heterozygous mutation of *Pcl* or *Su(z)12* mitigates PR cell loss. Heterozygous reduction of *E(z)* also mitigates PR loss. Scale bar indicates 10 μm. **b** Histogram showing the distribution of photoreceptors (PR) per ommatidium at 21d in wild-type (black, 7.0 PR/ommatidium), SCA3trQ78 (red, average 1.72 ± 0.06 SEM noted with red arrowhead, *n* = 24 flies, 240 ommatidia), and SCA3trQ78 with a heterozygous *Pcl* allele (green, average 4.02 ± 0.10 SEM noted with green arrowhead, *n* = 20 flies, 200 ommatidia). *p* < 0.001, Kruskal–Wallis test, Dunn post-test. Genotypes: SCA3trQ78 is *rh1-GAL4, UAS-SCA3trQ78/*+; *Pcl*^*5*^/+ is *Pcl*^*5*^*/*+; *rh1-GAL4, UAS-SCA3trQ78/*+. **c** Histogram showing the distribution of photoreceptors (PR) per ommatidium at 21d in wild-type (black, 7.0 PR), SCA3trQ78 (red, average 1.92 ± 0.06 SEM noted with red arrowhead, *n* = 29 flies, 291 ommatidia), and SCA3trQ78 heterozygous for a *Su(z)12* allele (green, average 4.93 ± 0.11 SEM noted with arrowhead, *n* = 20 flies, 197 ommatidia). *p* < 0.001, Kruskal–Wallis test, Dunn post-test. Genotypes: SCA3trQ78 is *rh1-GAL4, UAS-SCA3trQ78/*+; *Su(z)12*^*2*^/+ is *rh1-GAL4, UAS-SCA3trQ78/Su(z)12*^*2*^. **d** Histogram showing the distribution of photoreceptors (PR) per ommatidium at 21d in wild-type (black, 7.0 PR), SCA3trQ78 (red, average 1.86 ± 0.06 SEM noted with red arrowhead, *n* = 19 flies, 379 ommatidia), and SCA3trQ78 heterozygous for an *E(z)* allele (green, average 3.44 ± 0.16 SEM noted with arrowhead, *n* = 19 flies, 349 ommatidia). *p* < 0.001, Kruskal–Wallis test, Dunn post-test. Genotypes: SCA3trQ78 is *rh1-GAL4, UAS-SCA3trQ78/*+; *E(z)*^*731*^/+ is *rh1-GAL4, UAS-SCA3trQ78/E(z)*^*731*^

Expression of a pathogenic form of human ATXN3 with an expanded polyQ repeat (SCA3trQ78) in photoreceptor cells of the fly eye induces age-dependent neural loss that can be readily visualized by the pseudopupil imaging technique (Fig. 5a)^32^. Whereas flies normally retain 7 ± 0 (mean, ± SEM) photoreceptors (PR) per ommatidial unit with age, expression of pathogenic SCA3trQ78 results in age-dependent photoreceptor loss to 1.72 ± 0.06 PR/ommatidial unit in flies aged 21d (Fig. 5a). Given that our data indicate that *Pcl* and *Su(z)12* are targets of *miR-34*, we hypothesized that decreasing the activity of these genes may produce effects similar to upregulation of *miR-34* to suppress polyglutamine-mediated neurodegeneration^6^. To assess the impact of these PRC2 components, we reduced their levels by introducing one copy of the null mutations *Su(z)12*^*2*^ or *Pcl*^5^ into flies expressing SCA3trQ78. When *Pcl* activity was decreased, photoreceptor cell loss was strongly mitigated: while animals expressing SCA3trQ78 on its own had an average of fewer than two photoreceptors per ommatidium (1.72 ± 0.06) at 21d, animals expressing SCA3trQ78 and heterozygous for *Pcl*^*5*^ retained 4.02 ± 0.10 PR/ommatidial unit (Fig. 5a, b). Similarly, animals expressing SCA3trQ78 and heterozygous for the null allele *Su(z)12*^*2*^ retained 4.93 ± 0.11 PR/ommatidial unit (Fig. 5a–c). Several other alleles of *Pcl* and *Su(z)12* were also tested, and all protected from polyglutamine-mediated toxicity (Supplementary Figure 4). We also confirmed that *Pcl* and *Su(z)*12 mutations did not affect the steady-state level of the SCA3trQ78 mRNA (Supplementary Figure 4). These data indicate that reduction of *Pcl* or *Su(z)12* function mitigates polyglutamine degeneration.

### *Pcl* and *Su(z)12* mutations mitigate polyglutamine aggregation

**Fig. 6:**
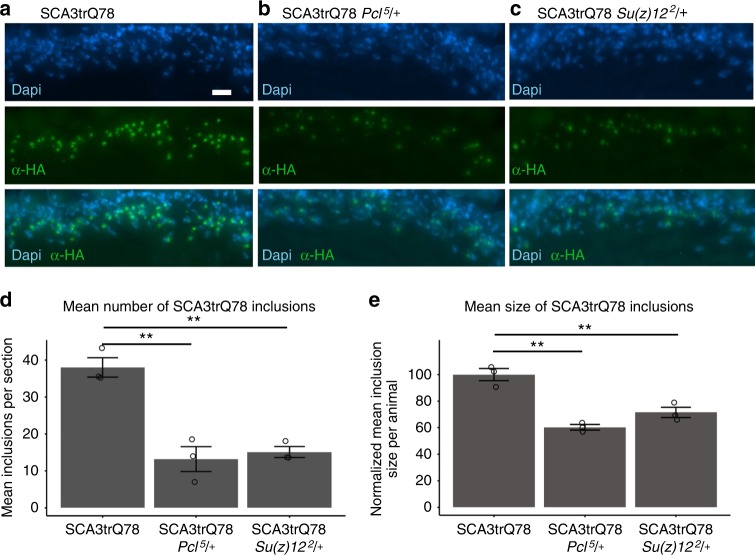
PRC2 mutations suppress accumulation of pathogenic polyglutamine protein. **a** Animals expressing SCA3trQ78 in the photoreceptor cells have prominent nuclear inclusions. 3d animals, antibody to HA tag present on the SCA3trQ78 transgene (green). Dapi (blue), nuclei. Genotype: *rh1-GAL4, UAS-SCA3trQ78/*+. Scale bar indicates 10 μm. **b** Decreasing *Pcl* activity decreased SCA3trQ78 inclusions of 3d animals. Genotype: *Pcl*^*5*^*/*+; *rh1-GAL4, UAS-SCA3trQ78/*+. **c** Decreasing *Su(z)12* activity decreased SCA3trQ78 inclusions of 3d animals. Genotype: *rh1-GAL4, UAS-SCA3trQ78/Su(z)12*^*2*^. **d** Average number of inclusions detected in the region of interest in various genotypes. Mean ± SEM, *n* = 3 animals, using the average of at least 4 sections per animal. F_2,6_ = 27.84, *p* < 0.001, one-way ANOVA, with Tukey post-test **p < 0.01. **e** Mean size of inclusions. The size of inclusions detected in at least 4 sections per animal was determined, with the control cohort adjusted to 100%. The average size of inclusions was smaller in animals heterozygous for *Pcl*^*5*^ or *Su(z)12*^*2*^. Mean ± SEM, *n* = 3 animals, using the average of at least 4 sections per animal. F_2,6_ = 30.89, *p* < 0.001, one-way ANOVA, with Tukey post-test, **p < 0.01

To investigate biological mechanisms through which genetic reduction of these PRC2 components impact neural degeneration, we conducted a detailed analysis of the aggregation of SCA3trQ78 protein in photoreceptor neurons. To assess inclusions, we examined tissue by immunohistochemistry. Normally, the polyglutamine protein accumulates rapidly, generating abundant, large nuclear inclusions. At 3d, many polyglutamine inclusions were visible in sections of the retinal neurons (38.0 ± 2.6) (Fig. 6a–d). When *Pcl* activity was reduced, the number of the inclusions decreased by 65%, to just 13.2 ± 3.4 (Fig. 6b–d). Additionally, there was a 39% decrease in the size of the remaining inclusions (Fig. 6e). Reduction of *Su(z)12* acted similarly, with the number of inclusions decreased by 60% to 15.1 ± 1.5 and the size of the remaining inclusions being smaller by 28% (Fig. 6c–e). These data suggest that the targets of the PRC2 complex that are critical to the effects of *miR-34* on polyglutamine toxicity may include genes that regulate protein misfolding and turnover, given these dramatic effects on protein aggregation and toxicity.

### Transcriptome profiling of the brains of *E(z)* mutants

As PRC2 functions as a histone modification complex, we anticipated that a consequence of higher PRC2 activity in aged *miR-34* mutants should be alterations in gene expression; the identification of these genes should include targets of the complex that are critical to healthy brain aging. For transcriptome analysis, we focused on mutants of the *E(z)* gene which encodes the catalytically active methyltransferase of PRC2: homozygous reduction of function *E(z)* mutants can be generated and should reflect the combined activities of reduction of both *Pcl* and *Su(z)12*. We confirmed that reduction of *E(z)* function mitigated polyglutamine toxicity (Fig. 5, Supplementary Figure 4). To reduce *E(z)* function, we generated animals heterozygous for a reported temperature-sensitive allele of *E(z)*, *E(z)*^61^, in *trans* to a null allele *E(z)*^*731*^. This allelic combination produced viable adult animals at the appropriate ratios at permissive (25 °C) and restrictive (29 °C) temperatures. Animals were cultivated and aged at either permissive or restrictive temperature, then brains were dissected and processed for RNA-seq analysis at timepoints of interest. We also prepared gene expression profiles of wild-type aged (20d) and young (3d) animals for comparison (details of samples and replicates in Supplementary Figure 5). We reasoned that comparing the *E(z)* reduction-of-function transcriptome to wild-type would reveal genes expressed in the brain that are normally regulated by PRC2 with age that might be modulated by *miR-34* function.

This approach identified 15,253 genes as being expressed in the brains of aged animals. Surprisingly, genes differentially expressed in *E(z)* reduction-of-function mutants compared to age-matched wild-type controls were differentially expressed at both the permissive and restrictive temperatures. This finding indicates that the *E(z)*^*61*^*/E(z)*^*731*^ transheterozygote combination has significant consequences for gene expression even at normal cultivation temperatures, consistent with our observations that mild removal of PRC2 activity mitigates polyglutamine toxicity (Fig. 5). We selected lists of genes differentially expressed using a Benjamini false discovery rate of *p* < 0.05, and a log_2_ fold-change of more than 0.5 (for upregulated genes) or less than −0.5 (for downregulated genes). This defined 343 upregulated and 290 downregulated genes upon reduced *E(z)* function (Supplementary Data 1). We examined GO Terms, Kegg Pathways, and InterPro motifs that were enriched in these lists of genes using DAVID^33^. Because we were interested in genes that would normally be targets of the H3K27me3 silencing mark and PRC2 is normally downregulated by *miR-34*, we first investigated genes that became upregulated with *E(z)* loss at 20d. GO terms associated with these genes included several relevant to age-associated processes, such as oxidoreductase activity (Supplementary Table 2). Additionally, the genes upregulated in *E(z)* mutants were enriched in the motifs Cytochrome P450 and Alpha-crystallin/sHSP family. We noted that the *Hsp70Ba* gene was also upregulated, which is a chaperone of the Hsp70 family, genes that are known to convey resistance to neurodegenerative disease^16,23,34,35^.

To define gene expression changes with age, we compared the transcriptomes of young and older wild-type brains, using a false discovery rate of *p* < 0.05 and an absolute log_2_ fold-change of more than 0.5. These criteria defined 672 downregulated and 959 upregulated genes (Supplementary Data 1). Again, due to our interest in genes that may normally be silenced by PRC2 with age, we focused on those genes that became downregulated with age in the brain. GO Terms associated with these genes included ATP synthesis coupled proton transport, among others (Supplementary Table 2). Notably, genes downregulated with age also included the Alpha-crystallin/sHSP category that was upregulated in the brain upon reduced function of *E(z)*.

**Fig. 7:**
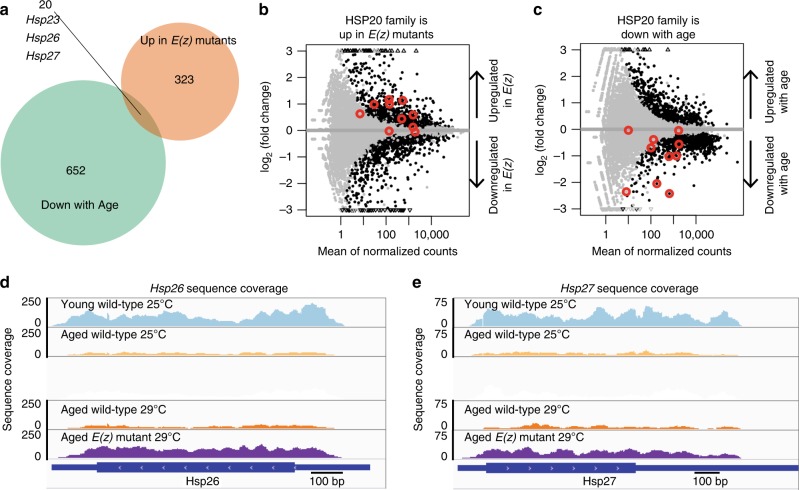
The small HSP chaperone family is regulated with age and by *E(z)*. RNA-seq was performed on 20d brain tissue from animals transheterozygous for the *E(z)*^*61*^ and *E(z)*^*731*^ alleles, and on wild-type. Because the *E(z)*^61^ allele is temperature-sensitive, two temperatures were used, 25 °C (permissive) and 29 °C (restrictive). Wild-type brains, 3d (young) and 20d (aged) 25 °C, were also dissected and RNA-seq performed. Genes were selected as differentially expressed using a Benjamini-Hochberg *p* < 0.05 and at least a 0.5 absolute log_2_ fold-change. **a** Gene overlap. Twenty genes had an expression pattern consistent with being a target of *E(z)* with age in the brain: upregulated in *E(z)* and downregulated with age. **b**, **c** Differential gene expression analysis of sHSP family genes. Gray data points are genes not differentially expressed. Black data points are genes with significant changes in expression (*p*<0.05, DEseq2 negative binomial generalized linear model, Benjamini-Hochberg correction).  Red circles mark the sHSP family members *Hsp23, Hsp26, Hsp27, Hsp67Ba, Hsp67Bc, l(2)efl, CG4461, CG7409, CG13133*, and *CG14207*. *Hsp22* was not included because no reads mapped uniquely to this gene. **b** Small HSP genes are upregulated in 20d *E(z)* mutant brains. Among these, *Hsp23, Hsp26, Hsp27*, and *CG7409* were significantly elevated in *E(z)* mutants (*p* < 0.05, DESeq2 negative binomial generalized linear model, Bonferroni correction), compared to 20d wild-type. **c** Small HSPs are downregulated in the brain with age. *Hsp23, Hsp26, Hsp27, Hsp67Ba*, and *Hsp67Bc* were significantly decreased (*p* < 0.05, DESeq2 negative binomial generalized linear model, Bonferroni correction) with age comparing 20d to 3d wild-type. **d**, **e** Sequence coverage at specific loci indicates silencing of sHSPs with age, and release from silencing in *E(z)* mutants. Read coverage at the individual sHSP loci (**d**) *Hsp26* and (**e**) *Hsp27* demonstrates a decrease in expression of sHSPs with age. Young wild-type animals express high levels (blue) while aged wild-type animals have less sHSP expression (yellow). *E(z)* mutation (purple) results in increased sHSP levels compared to age and temperature matched controls (orange). Y-axis indicates sequence read coverage from one biological replicate from each cohort. Each library shown had similar depth of sequencing (24–26 × 10^6^ reads). X-axis indicates position along the gene. Expression levels in *E(z)* are high, similar to young brain tissue

We expected that target(s) of PRC2 relevant to healthy brain aging would be genes that normally become downregulated with age in the wild-type brain. We therefore compared the overlap of genes, GO Terms, Kegg Pathways, and InterPro motifs among genes that are downregulated with age to those that are upregulated in the *E(z)* mutant. This identified four categories: GO:0005887∼integral component of the plasma membrane, dme:01100:Metabolic pathways, IPR002068:Alpha crystallin/Hsp20 domain, and IPR001436:Alpha crystallin/Heat shock protein.   Intriguingly, sHSPs were common to both sets of genes (Fig. 7a, Supplementary Figure 5, Supplementary Table 2). This overlap is particularly noteworthy for protein aggregation processes, as this family of small chaperones has been reported to mitigate polyglutamine toxicity and extend lifespan^17,18,36^. Five alpha-crystallin/sHSP genes were upregulated in *E(z)* mutants: *Hsp23, Hsp26, Hsp27, CG13133*, and *CG7409*. An MA plot of the ten HSP20 family members expressed in brain showed a strong bias towards higher expression in *E(z)* mutant brain tissue (Fig. 7b). The same HSP20 family members were also strongly downregulated with age (Fig. 7c). Sequence coverage is illustrated for *Hsp26* and *Hsp27* in Fig. 7d, e, which shows that expression in the aged *E(z)* brain is similar to that of the young brain profile, whereas normally their expression becomes downregulated with age. We confirmed several gene expression changes using qPCR. The sHSPs *CG7409*, *Hsp23*, *Hsp26*, *Hsp67Ba*, and *Hsp67Bc* were significantly upregulated in *E(z)* mutant brains, as was *Hsp70* (Supplementary Figure 5, Supplementary Table 3).

### The *E(z)* mutant brain shows a youthful transcriptome

*MiR-34* mutant brains have a transcriptional profile consistent with advanced age beyond their chronological age^6^. To determine this, we had defined 173 probe sets as highly correlated with age in the brain using linear regression statistical methods^37^ and then the transcriptional profile of the *miR-34* mutant brain was assessed for the levels of these probes to determine whether the mutant displayed the same, a more advanced or a less advanced age. The genes corresponding to these probesets define a “measuring stick” of brain age, to which we could compare our current transcriptomic data. We thus used the genes corresponding to these probesets to determine whether the *E(z)* mutant brain also impacted the transcriptional profile in a way that indicated an altered age. Because *miR-34* mutants display a premature-aged gene expression profile, we considered that *E(z)* brain tissue may correspond to a younger age profile, if targets of PRC2 relevant to aging of the brain are impacted.

**Fig. 8:**
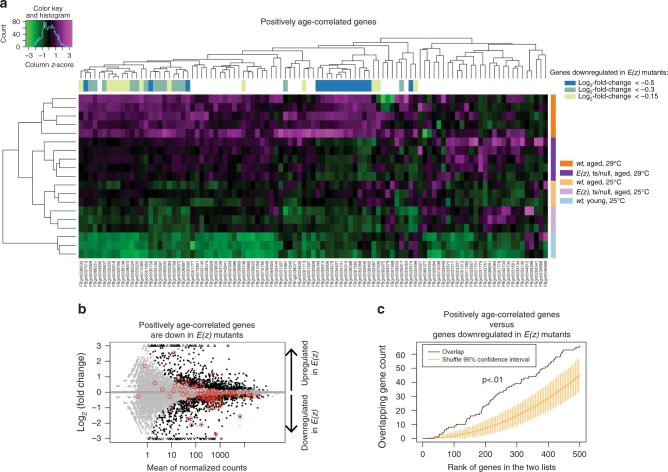
*E(z)* mutants have a chronologically younger aged transcriptome. **a** Heatmap of positively age-correlated genes. A set of genes previously shown to be positively correlated with age in the *Drosophila* brain^6^ were analyzed using hierarchical clustering to assess their pattern of expression in the *E(z)* mutant combination. Magenta indicates high and green indicates low expression. Colored bars on the side indicate genotype, age, and temperature. Colored bars on the top refer to genes downregulated in *E(z)* mutants, with color indicating the log_2_-fold-change threshold. Brains from young animals have the lowest expression of positively age-correlated genes, followed by brains from 20d *E(z)* mutants. Brains from 20d wild-type animals cultivated at 29 °C generally have the highest expression levels. For two clusters of genes (blue genes), expression is decreased in *E(z)* mutants relative to age-matched and temperature-matched wild-type controls. **b** Genes positively correlated with age are disproportionately downregulated in *E(z)* mutants. Gray data points are genes not differentially expressed. Black data points are differentially expressed genes (*p* < 0.05). Red circles are genes corresponding to probe sets previously defined as positively correlated with age^6^. The overlap between genes called downregulated in *E(z)* and the genes positively-correlated with age is statistically significant (*p-*value = 0.001, hypergeometric test). **c** Significant overlap between genes positively correlated with age and genes downregulated in *E(z)* mutants indicate a less advanced age transcriptome profile. Comparison of two ranked lists of genes: the list of genes ranked by degree of downregulation in *E(z)* mutants (Supplementary Data 1) and top 500 genes ranked by positive correlation with age Supplementary Data 2. *E(z)* regulated genes were ranked by the product of the -log(*p*-value) and the moderated log_2_ fold-change (low to high). All positively age-correlated genes were ranked according to decreasing significance of the correlation with age among all genes with a positive correlation, followed by genes with negative correlation which were ranked according to increasing significance (Supplementary Data 2). Yellow bars indicate 95% confidence intervals. By contrast, the correlation from the data (black line) is outside of the confidence intervals and thus is statistically significant (*p* < 0.01, calculated empirically using permutations, orderedList R-package)

Hierarchical clustering of the positively age-correlated measuring stick genes across the samples identified two clusters of genes that were downregulated in *E(z)* mutant brains (Fig. 8a, genes downregulated in *E(z)* mutants indicated with blue/green/yellow coloring for log_2_-fold-change magnitude, as noted in the legend on right). The genes downregulated in *E(z)* mutant brains exhibited significant overlap with the set of positively age-correlated genes (blue genes Fig. 8a, b, *p* < 0.001, hypergeometric test). This finding indicates that the *E(z)* mutant brain has a transcriptional profile of a brain of less advanced age.

We further addressed this question using the orderedList Bioconductor package^38^. This method avoids the use of arbitrary effect-size and significance cutoffs when probing for overlap in gene expression patterns. We compared the list of genes from the *E(z)* expression experiment, ranked by the product of the –log_10_ (*p* value) and the moderated log_2_ fold-change (low to high, such that genes downregulated in *E(z)* mutants are at the top), to the list prepared from the age-correlated genes that serve as a measuring stick for brain age (Supplementary Data 2), in which genes with most significant positive age correlations are ordered first, and genes with most significant negative age correlations are ordered last. These lists were then compared for statistically significant similarities. This approach showed that the overlap of these two lists was significant only for the positively-correlated genes (Fig. 8c), again indicating that *E(z)* mutants have a gene expression profile consistent with a less advanced age when using these genes for a measuring stick of brain age. No trend was discerned for probe sets negatively-correlated with age (Supplementary Figure 5e, f), as with the *miR-34* gene expression profile^6^.

Collectively, these findings indicate that *E(z)* reduction-of-function mutants have several gene expression characteristics that are conducive to healthy brain aging: an overall transcriptome signature of age consistent with a more youthful brain, as well as elevated sHSP chaperone expression, and elevated *Hsp70* expression.

## Discussion

We present an age-associated gene expression pattern of the brain that can be both accelerated (in the *miR-34* mutant, resulting a gene expression profile consistent with advanced age^6^), as well as decelerated, resulting in an expression profile consistent with a more youthful age (in the *E(z)* mutant, Fig. 8b, c). These gene expression changes are a result of manipulations of a naturally occurring regulatory circuit in which *miR-34*, which increases in expression in the adult brain with age, acts to help reduce translation of the PRC2 components *Su(z)12* and *Pcl*. In the absence of *miR-34*, Su(z)12 and Pcl protein levels are more elevated in the brain (Figs. 2, 3), and the PRC2 associated chromatin mark H3K27me3 is higher (Fig. 4). Decreasing the activity of components of PRC2 results in resistance to neurodegeneration and stress, and lifespan extension (Fig. 5)^13,14^. Importantly, gene expression profiling of mutants in the catalytic component of PRC2, *E(z)*, reveals decreases in expression of genes positively correlated with age (Fig. 8b, c). These experiments reveal that *miR-34* mutants and reduction in PRC2 activity have inverse effects on age-associated gene expression patterns in the brain: *miR-34* mutants have a gene expression profile of advanced age, whereas *E(z)* mutants have a gene expression profile of a younger brain age. Taken together, these data indicate that mitigation of the activity of the PRC2 complex leads to healthier brain aging and protection from degenerative disease.

Our studies suggest that Pcl and Su(z)12 are targets of -34 activity in the brain of the fly with age. We show that miR-34 can modulate the levels of these transcripts in luciferase assays (Fig. 1), and that their protein levels are altered in the brain with age (Figs. 2, 3). MiR-34 appears to function to make normal age-associated changes in these components more robust with age, a role that has been associated with miRNAs^39^. In our studies, the interaction between miR-34 and Su(z)12 appears stronger than Pcl, given the protein changes with age. Interestingly, potential miR-34 seed sites are present in vertebrate homologs of these genes *PHF19* and *SUZ12* (Fig. 1), raising the possibility that regulation by miR-34 may also occur in mammals, including humans. Consistent with this regulation, H3K27me3 levels become increased in the *miR-34* mutant brain with age (Fig. 4). These data suggest that miR-34 helps to modulate the activity of the PRC2 complex in the brain with age. We note that future studies using more directed mutations, with the use of Crispr/Cas9 could be helpful to fine tune the requirement for miR-34 seed sequences in the *Pcl* and *Su(z)12* genes.  As PRC2 is a histone methyltransferase that silences gene expression^25,40^, we used mutants in the catalytic component of the PRC2 complex *E(z)* as a more potent approach than reduction of *Pcl* and *Su(z)12*, to define potential genes modulated by PRC2 with age. The *E(z)* allelic combination was reduction of function, and not null, and revealed a transcriptomic profile of a brain of younger age (Fig. 8).

Among genes affected by the *E(z)* mutant were chaperones. Upregulated chaperone gene expression could explain many of the healthy age-associated effects of PRC2 reduction such as extension in lifespan, stress resilience, and the younger brain transcriptome profile. Small HSP proteins have been shown to suppress polyglutamine aggregation and extend lifespan^17,18,36^, which are two phenotypes associated with reduction of PRC2 components (Figs. 5, 6)^13,14^. Several sHSP proteins are also upregulated in the long-lived *daf-2* mutant in *C. elegans*^22^, also underscoring an association with healthspan. Chaperones of the Hsp70 class are also known to suppress neurodegeneration^16,23,35^, are upregulated in the brains of *E(z)* mutant animals and may contribute to the protective effects of PRC2 inhibition. In humans, mutations in various sHSPs are associated with age-associated neuropathies, making these proteins of interest for their role in the brain with age^41,42^.

**Fig. 9:**
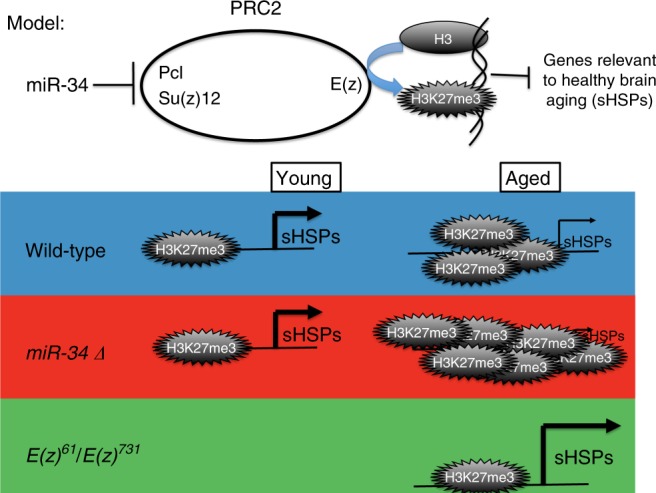
Model for opposing activities of *miR-34* and the PRC2 complex in aging. Our data suggest that *miR-34* represses translation of *Pcl* and *Su(z)12* transcripts, resulting in a reduction of PRC2 activity and less H3K27me3. In the *miR-34* mutant and normally with age, H3K27me3 accumulates globally and at particular genes, resulting in decreased transcription. Some of these genes (the small heat shock proteins (sHSPs) are highlighted here) appear critical for healthy brain aging. Mutation of *E(z)* reduces the activity of PRC2, resulting in less suppression of sHSPs and a younger brain transcriptome profile. These data indicate that reduction of the activity of PRC2 with age will be associated with healthier brain aging and protection from deleterious consequences to the brain with age, including neurodegenerative disease

We emphasize the importance of reduction of PRC2 function (seen in the allele combination employed here, and effected by *miR-34* modulation), versus elimination of PRC2 function in the brain. Mice with severe mutation for PRC2 activity in the striatum suffer from neurodegeneration^43^. In those experiments, PRC2 was conditionally inactivated using null mutations in both mouse homologs of *E(z)* (*EZH1* and *EZH2*). Although it was proposed from that finding that PRC2 activity could normally be protective against neurodegeneration^43^, null mutations in PRC2 component genes including mammalian *EZH2* and *SUZ12* are embryonically lethal^25,40,44–48^. Thus, critical cellular and/or developmental functions are provided by this complex. We suggest that null mutations will result in stronger and perhaps more gene expression changes that eliminate critical functions, compared to the situation of reduction—but not elimination—of PRC2 activity achieved in our studies and by *miR-34*. Consistent with this latter interpretation, in a disease model for ataxia telangiectasia, shRNA knock-down of *EZH2* was protective against neurodegeneration^15^. We propose a model whereby partial reduction of PRC2 activity results in healthier brain aging (Fig. 9), whereas null mutation confers pleiotropic negative effects leading to extreme consequences.

Our studies associate the *miR-34/*PRC2 axis as important for regulating gene expression patterns associated with brain aging. Our data suggest that this axis can be genetically manipulated to mimic both a more advanced age, as well as a more youthful age profile. We can imagine several ways by which decreased PRC2 activity promotes healthy brain aging. In one model, PRC2 could act as a master regulator to silence numerous genes that promote healthy brain aging. Although we have highlighted a set of genes that may be of special importance, many genes or even genome wide effects may contribute to the outcome. Alternatively, PRC2 could suppress the expression of a select, but critical, set of genes with great importance to long term health of the brain, such as chaperones and/or oxidoreductases. By this scenario, the corresponding healthy brain transcriptome pattern is a downstream effect of elevated activity of a small set of genes. Identification of the specific genes that are targeted by H3K27me3, followed by functional implication of these genes, may discriminate between these models.

Intriguingly, our studies raise the possibility that genes of the stress response, reflected here by molecular chaperones, may be a target of epigenetic silencing in the brain with age. The outcome would be compromised resilience to stress, resulting in neurodegeneration. Our discovery of the PRC2 complex as a key modulator of a healthy brain transcriptome in *Drosophila* may extend to the vertebrate brain, as *miR-34* seed sequences are present in the 3′UTRs of vertebrate homolog of *Pcl*, *PHF19*. Small molecules that target epigenetic modulation of the genome are in development as cancer therapeutics^49,50^. These findings thus raise the possibility that mitigating PRC2 activity, through gene or small molecule approaches, may lead to healthier brain aging and protection from associated deleterious consequences, such as cognitive decline and neurodegenerative disease.

## Methods

### Fly strains

*Drosophila* were maintained at 25 °C, unless otherwise indicated, and raised on standard molasses fly meal. For GeneSwitch experiments, 50 ul ethanol (vehicle control) or 50 uL 4 mg/ml RU486 dissolved in ethanol was added to food vials and allowed to dry with gentle rocking for 24 h. *E(z)* alleles were backcrossed at least five times to the homogeneous *w*^*1118*^ stock (Bloomington line 5905) wild-type control, selecting for heterozygous animals using restriction fragment length polymorphisms (RFLP), (Supplementary Table 4). Male flies were used for all experiments. Complete genotypes and cross details of flies used are provided (Supplementary Table 7).

### Brain dissections

In many experiments, brains were carefully dissected from the head capsule for use due to enrichment of *miR-34*. Flies were mildly anesthetized using CO_2_ and decapitated using a razor blade. The head was placed posterior side down and the proboscis was then removed using Dumont #5SF forceps (Fine Science Tools, Foster City, CA). The head was then simultaneously squeezed from both lateral sides and the posterior resulting in slight protrusion of the brain through the proboscis cavity. Forceps were then used to drag it out of the head capsule and place it in ice-cold PBS in a glass dissection well. Further dissection of remaining eye and trachea tissue was under cold PBS. Brains were then transferred to a microfuge tube using a Pasteur pipet, PBS was aspirated, and brains were ground in Laemmli Buffer (1–2 μL per brain, at least 20 brains for each sample) for Western immunoblotting, or Trizol for RNA-seq analysis (see below).

### Luciferase assays

All primers for constructs used in these experiments are listed in Supplementary Table 5. *Su(z)12* luciferase reporters were constructed using PCR primers to amplify 953 nucleotides of the 3′UTR from *Su(z)12*. The resulting fragment was cloned into the *Bam*HI/*Sal*I sites in pMT-Renilla. The *Pcl* 3′UTR was prepared using overlap extension of synthesized oligos utilizing Klenow fragment to produce a 211 bp 3′UTR which was cloned into the *Bam*HI/*Sal*I sites in pMT-Renilla. Mutations were induced to disrupt the seed sequence using the Quikchange mutagenesis system (Stratagene, La Jolla, CA).

For the luciferase reporter assay, cells were plated at 1 × 10^6^ cells/mL, in a total of 2 ml for each transfection in a six-well plate. Cell lines from the *DGRC*. Transfection of pMT-Renilla luciferase reporter containing the 3′UTR of interest, a transfection efficiency marker (pMT-Firefly), and a plasmid upregulating *miR-34* (pMT-*miR-34*) or a control (pMT-GFP), was performed the next day using Effectine (Qiagen, Germantown, MD). For SL2 cells, amounts of plasmids transfected per well were 100 ng, 40 ng, and 2 μg, respectively. For DL1 cells, amounts used were 1067 ng pMT-Renilla reporter, 4 ng pMT-Firefly, and 1067 ng pMT-*miR-34* or pMT-*miR-277* control. 1 day after transfection of SL2 cells, the cells were pelleted, resuspended in 5.5 mL complete media and distributed to a 96-well plate, 150 μL in each well. 1 day after re-plating, CuSO_4_ was added to a final concentration of 1 mM. 1 day later, Firefly and Renilla luciferase levels were measured using Dual-Glo Luciferase Assay System (Promega, Madison, WI) with an Analyst HT luminometer.

For the dsRNA experiments in DL1 cells, 1 day after transfection cells were instead treated with dsRNA to the *AGO1* gene, or an *E. coli beta-galactosidase* control (previously described^51^). For dsRNA treatment, cells were harvested, pelleted, and resuspended in 5.5 mL serum-free media. DsRNA (0.75 ng) was placed in each well of a 96-well plate, and 50 μL of cells were added. After 45 min, 100 μL of complete media was added to each well. 3 days later, CuSO_4_ was added to 1 mM, and the next day luciferase levels were measured as above.

### Western immunoblotting

Conditions were optimized for each antigen/antibody pair. Antibodies and conditions are listed in Supplementary Table 6. HRP signal was developed using ECL (Amersham, Piscataway, NJ) and imaged on an Amersham Imager 600. Histones derived from heads were purified using Histone Purification Mini Kit (Active Motif, Carlsbad, CA). Quantification of bands was performed with ImageJ^52^. Uncropped Western images with size markers are presented in Supplementary Fig. 6.

### Su(z)12-HA-tag construction

One copy of the HA tag sequence, YPYDVPDYA, was fused in frame to the coding region of *Su(z)12* just prior to the endogenous stop codon, followed by a *Bam*HI cloning site. Fragments of the *Su(z)12* 3′UTR derived from the luciferase reporters were cloned into pCaSpeR4, one with a wild-type 3′UTR, and one with the mutated seed 3′UTR. The promoter and coding sequence were amplified using primers listed in Supplementary Table 5, the fragment was then cloned into the pCaSpeR4 vectors already containing the wild-type 3′UTR from *Su(z)12*, and the mutated 3′UTR from *Su(z)12* to produce Su(z)12-Cterm-HA (pJRK228) and Su(z)12-Cterm-HA-NsiI (pJRK235).

### Pseudopupil assay

Flies were mildly anesthetized using CO_2_ and decapitated using a razor blade. Heads were mounted onto a microscope slide to allow illumination from the posterior along the axis of the ommatidia, resulting in visualization of photoreceptors. Heads were positioned using a thin line petroleum jelly, positioning the head in the jelly such that the light path travels through a minimum of head tissue, and does not travel through petroleum jelly. The head was then covered in immersion oil suitable for an oil immersion objective (100×). The photoreceptors are then visible with bright field imaging, with the condenser adjusted for high intensity, highly focused light centered beneath the ommatidia in the field of view. Experiments were done with blinding to genotype.

### Immunocytochemistry

Flies were mildly anesthetized using CO_2_ and decapitated using a razor blade. The head was placed posterior side down and the proboscis and air sac were removed using forceps. The head was then placed on Tissue Freezing Medium (TFM) (Electron Microscopy Sciences, Hatfield, PA), and the proboscis cavity was filled with TFM. The head was then submerged and any bubbles were removed. The head was oriented inside an Embedding Mold (Polysciences, Warrington, PA) to allow for coronal sections, and frozen in a dry ice/ethanol bath. Fly heads were then cut to 12μ sections on a CM3050S cryostat (Leica, Buffalo Grove, IL). Sections were placed on Colorfrost Plus Microscope Slides (Fisher Scientific, Pittsburgh, PA), and kept at −80 °C until all replicates were finished.

Frozen sections were then warmed to 37 °C for 30 min. Sections were outlined with a PAP pen (Daido Sangyo Co., Tokyo, Japan). Sections were fixed in 0.5% paraformaldehyde in phosphate buffered saline (PBS) for 30 min at room temperature (RT), then washed 3 times for 5 min each in PBS. Sections were blocked in PBSG (1% goat serum, 0.2% Bovine Serum Albumin, and 0.01% saponin in PBS) for 1 h at RT, followed by incubation with primary antibody (rat anti-HA 3F10, Roche 11867423001, at 1:20 dilution in PBSG) overnight in a humidified chamber at 4 °C. Primary antibody stained sections were then washed 3 times for 5 min each in PBSG, followed by secondary antibody (Affinipure goat anti-Rat conjugated FITC, 1:20 dilution in PBSG, Jackson ImmunoResearch) incubation for 1 h at RT. Sections were then rinsed 3 times for 5 min each in PBS, followed by staining with Hoechst 33342 stain (Molecular Probes) for 10 min, followed by 3 more PBS washes for 5 min each. Sections were then covered in vectashield mounting medium (Vector Laboratories, Burlinggame, CA), and a coverslip was applied.

### Statistics

Statistical tests were done in R or with Prism (GraphPad Software, La Jolla, CA). One-way or two-way ANOVA was performed followed by Tukey′s post hoc test for multiple comparisons. For each ANOVA, the Shapiro-Wilk test^53^ was performed to test for non-normality in the data, and the Levene test^54^ was performed to test for heterogeneity of the variance. No statistical methods were used to choose sample size. No biological sample replicates were excluded. Each biological replicate indicates animals derived from distinct genetic crosses. No randomization methods were used.

### RNA sequencing

Once at least 20 brains were collected as described above, brains were transferred to an Eppendorf tube and homogenized in 50 μL Trizol (Thermo Fisher Scientific, Waltham, MA) using a pellet pestle. An additional 200 μL of Trizol was then added and the tissue frozen at −80 °C. RNA was prepared using the manufacturer recommended procedure for Trizol reagent. Any residual DNA was removed using DNA-free DNAse digestion (Ambion, Grand Island, NY). Total RNA was then cleaned up using RNAeasy columns (Qiagen, Germantown, MD). The quality of the resulting mRNA was validated using a Bioanalyzer and quantified with Qubit. 95–200 ng of total RNA was used for TruSeq stranded mRNA library preparation according to the manufacturer’s protocol (Illumina, San Diego, CA). Libraries were quantified using both Qubit and Kapa Quantification systems (Kapa Biosystems, Wilmington, MA) and pooled accordingly. Libraries were run on two NextSeq500 flow cells (Illumina, San Diego, CA) at 75 nucleotide sequence read length.

### RNA-seq data analysis

Reads were mapped to the *Drosophila* Genome r6.15 with STAR using the default parameters^55^. Reads were assigned to exons in the FlyBase version6.15 GTF file using the htseq-count Python package^56^. Reads per gene were then analyzed using the DESeq2 R package from Bioconductor^57^. Gene expression changes observed in *E(z)* mutants were not restricted to animals cultivated at the restrictive temperature of 29 °C: only three genes showed a temperature-dependent gene expression change consistent with the reported temperature-sensitive nature of the allele, as indicated by testing for a significant interaction of temperature and genotype. As such, genes upregulated and downregulated in 20d *E(z)* mutant brains relative to 20d wild-type were selected using a two-way statistical model including both temperature and genotype as independent categorical variables. When determining log_2_ fold change and *p* values of wild-type vs. *E(z)* mutant, the comparison was made using both 25 °C and 29 °C samples; blocking on temperature was used to determine gene expression changes that were a consequence of *E(z)* mutation. Statistical significance was determined using the Wald test provided as part of the DESeq2 package.

Genes upregulated and downregulated with age were selected by comparing 20d wild-type animals cultivated at 25 °C to 3d wild-type animals, also cultivated at 25 °C. Expression differences were determined using age as the independent categorical variable modeled with DESeq2 using the Wald test for statistical significance.

Differentially expressed genes were selected using the maximum *a posteriori* (MAP) absolute log_2_ fold change of at least 0.5 as calculated by the DESeq2 package and a Benjamini-Hochberg adjusted *p* value of < 0.05. The lists of differentially expressed genes were used for DAVID GO analysis^33^, comparing to the background list of all genes whose expression level in brain was above the threshold for significance testing, as determined by the DESeq2 package. Age-correlated probesets^6^ were translated to Ensembl genes using the biomaRt R package^58^. Only probesets that overlapped genes expressed in brain by the RNA-seq data were used in the analysis. This, along with redundancy in some of the probesets, resulted in condensing the 108 positively age-correlated probesets to 102 ENSEMBL genes (Supplementary Data 2). RNA-seq data and count matrices are accessible under Gene Expression Omnibus (GSE110135).

### Code availability

The R code used for all analyses is available upon request.

## Electronic supplementary material

Supplementary Information

Description of Additional Supplementary Files

Supplementary Data 1

Supplementary Data 2

## Data Availability

The datasets generated during the current study are available under accession GSE110135 in NCBI GEO (https://www.ncbi.nlm.nih.gov/geo/query/acc.cgi?acc = GSE110135) and upon request.
