# Supplementary material for: MiR-34 inhibits polycomb repressive complex 2 to modulate chaperone expression and promote healthy brain aging
Source: Nat Commun. 2018 Oct 10;9:4188. doi: 10.1038/s41467-018-06592-5 (PMC6180074; doi:10.1038/s41467-018-06592-5)
Supplement: Supplementary file 1 — Supplementary Information [file 41467_2018_6592_MOESM1_ESM.pdf]

Supplemental material to:

MiR-34 inhibits Polycomb Repressive Complex 2 to modulate chaperone expression and promote healthy brain aging

Jason R. Kennerdell[1], Nan Liu[1,2], and
Nancy M. Bonini[1*]

[1]Department of Biology, University of Pennsylvania, Philadelphia, PA 19104

[2] Current Address: Interdisciplinary Research Center on Biology and Chemistry, Shanghai Institute of Organic Chemistry, Chinese Academy of Sciences, Shanghai, 201210 China

[*]Corresponding Author

SUPPLEMENTARY METHODS

Realtime qPCR:
RNA was purified from dissected brains using Trizol, followed by DNA-free treatment (Ambion, Waltham, MA), followed by RNeasy cleanup (Qiagen, Germantown, MD). Purified RNA (20ng) was used to generate cDNA using the High Capacity cDNA Reverse Transcription kit (Applied Biosystems, Waltham, MA) in a reaction of 10μL. cDNA was diluted 100–fold and 4.5 μL was used as template in each realtime PCR reaction with 5μL FAST SYBR Master Mix and 0.5μL 1μM primers. Reactions were run in a ViiA 7 Realtime PCR system. Quantification was done using the ΔΔCt method, normalizing to the geometric mean of three endogenous controls (*RpS20*, *pgk*, and *tbp*). Primers, with validation parameters for all amplicons, are listed in SupplementaryTable 3. Data and statistics were analyzed in R.

SUPPLEMENTAL REFERENCES

1. Liu N*, et al.* The microRNA *miR-34* modulates ageing and neurodegeneration in *Drosophila*. *Nature.* **482**, 519-523 (2012).

2. Paraskevopoulou MD*, et al.* DIANA-microT web server v5.0: service integration into miRNA functional analysis workflows. *Nucleic Acids Res.* **41**, W169-173 (2013).

3. Enright AJ, John B, Gaul U, Tuschl T, Sander C, Marks DS. MicroRNA targets in Drosophila. *Genome Biol.* **5**, R1 (2003).

4. Grun D, Wang YL, Langenberger D, Gunsalus KC, Rajewsky N. microRNA target predictions across seven Drosophila species and comparison to mammalian targets. *PLoS Comput Biol.* **1**, e13 (2005).

5. Ruby JG, Stark A, Johnston WK, Kellis M, Bartel DP, Lai EC. Evolution, biogenesis, expression, and target predictions of a substantially expanded set of *Drosophila* microRNAs. *Genome Res.* **17**, 1850-1864 (2007).

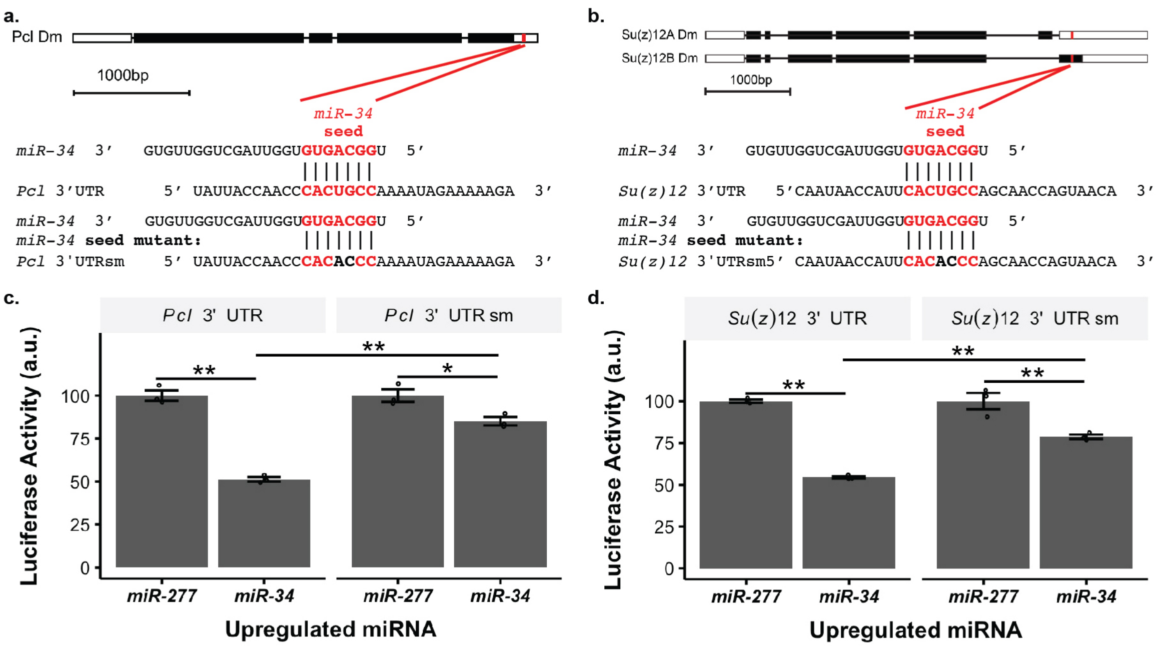

Supplementary Figure 1: Regulation of *Pcl* and *Su(z)12* 3’UTRs using *miR-277* as control (Associated with Figure 1)

a, b. A mutation of two nucleotides (UG to AC) was introduced into the 3’UTR of the *Pcl* gene (**a**), so as to disrupt base-pairing between *miR-34* and the *Pcl* transcript, as well as the (**b**) *Su(z)12* 3’UTR. Seed sequence indicated in red, disruptions indicated in black.

c, d. Luciferase assays confirm that *miR-34* targets the *Pcl* and *Su(z)12* 3’UTRs. A reporter construct containing the 3’UTR of (**c**) *Pcl*, or (**d**) *Su(z)12* fused to Renilla luciferase was expressed in *Drosophila* DL1 cells and tested for a response to *miR-34* upregulation. As a control, upregulation of an unrelated miRNA, *miR-277*, was used. Upon upregulation of *miR-34*, luciferase activity decreases in reporters containing the (**c, left**) *Pcl* 3’UTR as well as the (**d, left**) *Su(z)12* 3’UTR. Downregulation of both 3’UTRs was attenuated (**c, d, right**) by a seed sequence mutation (**a, b**). Two-way ANOVA indicated a significant interaction term for the *Pcl* 3’UTR ($F_{1,8}$=28.7, p<.001), and *Su(z)12* 3’UTR ($F_{1,8}$=28.5, p<.001). *p<.05, **p<.01, two-way ANOVA with Tukey post-test. Mean ± SEM, n=3 wells.

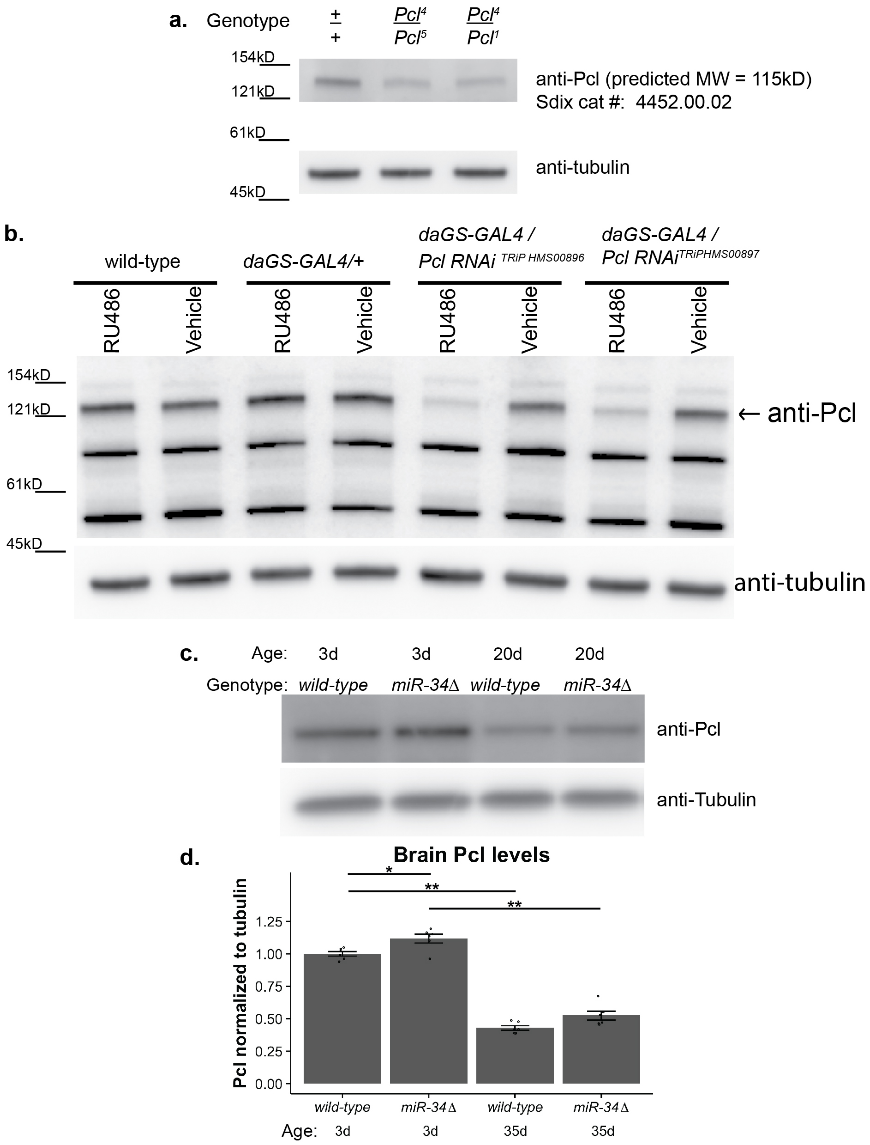

Supplementary Figure 2: Validation of Pcl antibody and extended timepoint (Associated with Figure 2)

a. Confirmation of the specificity of the Pcl antibody (Sdix, Newark, DE). Three bands were detected; however, only one was decreased in animals with mutations in *Pcl*. Two genotypes were tested, *Pcl^4/Pcl^5* and *Pcl^4/Pcl^1*, and a wild-type control +/+. Adult male brains, 3d of age.

b. Specificity was also confirmed using knock-down of *Pcl*. Adult flies of the indicated genotypes were aged in the presence of RU486 or vehicle control upon eclosion from the pupal case. RU486 induces activity of GAL4 Geneswitch under the control of the constitutive *daughterless* promoter, inducing *UAS-Pcl RNAi*. Brains were dissected from adult males 10d of age. Tubulin, loading control.

c. Pcl protein levels are de-regulated in *miR-34* mutant brains (extended timepoint). Western immunoblots against Pcl (top) and Tubulin (loading control, bottom).

d. Quantitation of immunoblots. Pcl levels decrease with age in wild-type and in *miR-34* mutant brains. Protein samples from dissected brains. Western immunoblot, with tubulin as the loading control. Pcl protein levels normalized to tubulin. Mean ± SEM, n=6 biological replicates. Significant main effects were observed (genotype: $F_{1,20}$=15.5, *p*<0.01 age: $F_{1,20}$=461.9, *p*<0.001). (**p*<0.05, ***p*<0.01, two-way ANOVA with Tukey post-test).

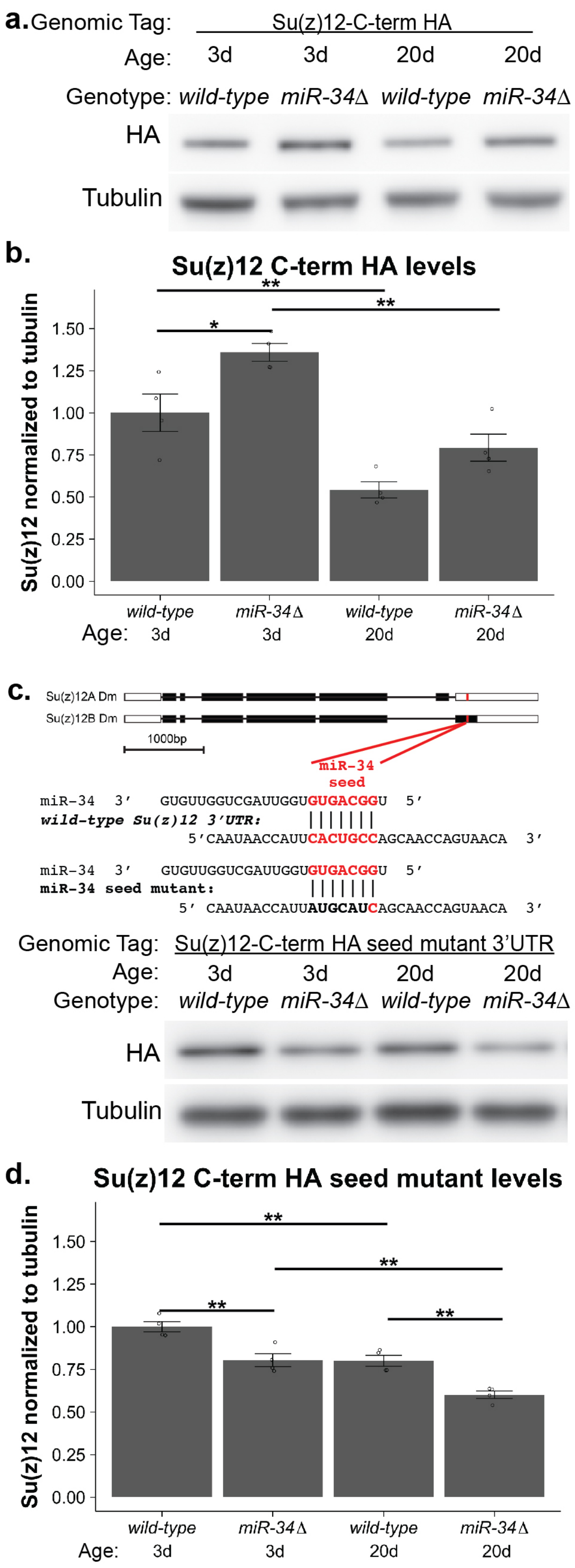
a.
Genomic Tag: Su(z)12-C-term HA
Age: 3d 3d 20d 20d
Genotype: wild-type miR-34Δ wild-type miR-34Δ
HA
Tubulin
b.
Su(z)12 C-term HA levels
Su(z)12 normalized to tubulin
1.50
1.25
1.00
0.75
0.50
0.25
0.00
**
**
*
wild-type miR-34Δ wild-type miR-34Δ
Age: 3d 3d 20d 20d
c.
Su(z)12A Dm
Su(z)12B Dm
1000bp
miR-34
seed
miR-34 3' GUGUUGGUCGAUUGGUGUGACGGU 5'
wild-type Su(z)12 3'UTR:
5'CAAUAACCAUUCACUGCCAGCAACCAGUAACA 3'
miR-34 3' GUGUUGGUCGAUUGGUGUGACGGU 5'
miR-34 seed mutant:
5' CAAUAACCAUUAUGCAUCAGCAACCAGUAACA 3'
Genomic Tag: Su(z)12-C-term HA seed mutant 3'UTR
Age: 3d 3d 20d 20d
Genotype: wild-type miR-34Δ wild-type miR-34Δ
HA
Tubulin
d.
Su(z)12 C-term HA seed mutant levels
Su(z)12 normalized to tubulin
1.50
1.25
1.00
0.75
0.50
0.25
0.00
**
**
**
**
wild-type miR-34Δ wild-type miR-34Δ
Age: 3d 3d 20d 20d

Supplementary Figure 3: Independently integrated reporter transgenes confirm that Su(z)12 is a target of *miR-34* in the brain (Associated with Figure 3)

a, b. Su(z)12 protein levels are de-regulated in *miR-34* mutants. Su(z)12 protein was detected using a transgene containing the *Su(z)12* genomic region tagged with an HA epitope on the C-terminus of the predicted protein (Su(z)12-C-term HA). **a.** Western immunoblotting to detect Su(z)12 reporter levels in dissected brain. **b.** Quantification of Su(z)12 levels. Su(z)12 reporter expression decreased with age in wild-type, but is elevated in *miR-34* mutants. Protein samples from dissected brains. Su(z)12-C-term HA protein levels were normalized to tubulin loading control. Mean ± SEM, n=4 biological repeats. Significant main effects were observed (genotype: $F_{1,12}$=15.5, p<0.002 age: $F_{1,12}$=43.9, p<0.001). (*p<0.05, **p<0.01, two-way ANOVA with Tukey post-test).

c, d. The regulation of Su(z)12 protein levels by *miR-34* is dependent upon the *miR-34* seed sequence in the 3'UTR of the *Su(z)12* transcript. The Su(z)12-C-term HA-mutant 3'UTR transgene contains a mutation in the *miR-34* seed sequence that relieves the transcript from regulation by *miR-34* (see Fig. 1d). **c.** The seed mutant is designed to disrupt base pairing between the Su(z)12 3'UTR of the transgene and miR-34. Su(z)12-C-term HA-mutant 3'UTR protein levels still decrease with age, but are no longer increased in *miR-34* mutants, and are in fact decreased relative to wild-type. Protein samples from dissected brains. **d.** Quantification of Su(z)12-C-term HA-mutant 3'UTR protein levels using Western immunoblotting, normalized to tubulin loading control. Mean ± SEM, n=4 biological replicates. Significant main effects were observed (genotype: $F_{1,12}$=40.4, p<0.001 age: $F_{1,12}$=41.5, p<0..001). (*p<0.05, **p<0.01 two-way ANOVA with Tukey post-test).

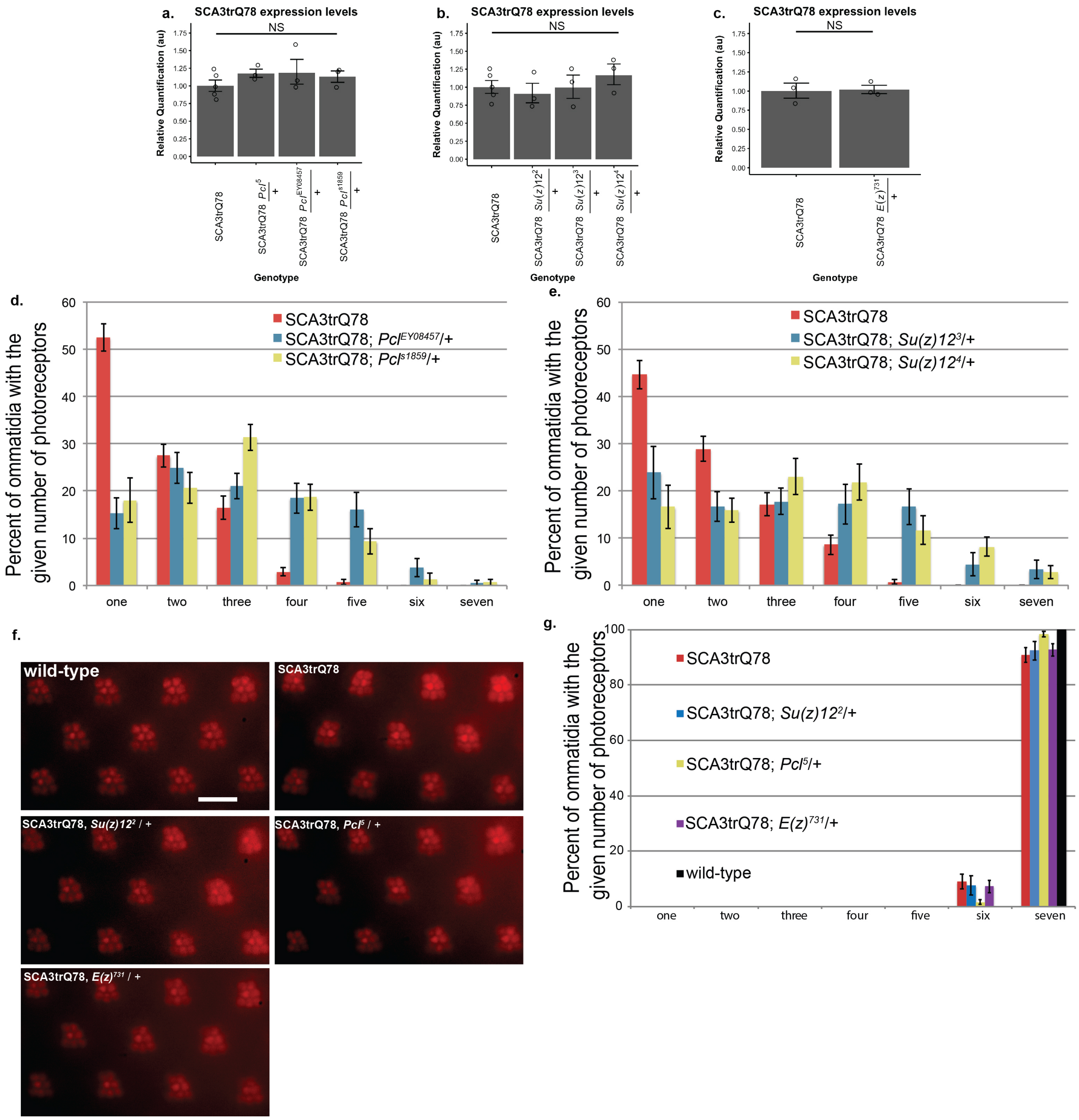
a.
SCA3trQ78 expression levels
NS
Relative Quantification (au)
Genotype
b.
SCA3trQ78 expression levels
NS
Relative Quantification (au)
Genotype
c.
SCA3trQ78 expression levels
NS
Relative Quantification (au)
Genotype
d.
Percent of ommatidia with the given number of photoreceptors
SCA3trQ78
SCA3trQ78; PclEY08457/+
SCA3trQ78; Pcls1859/+
one two three four five six seven
e.
Percent of ommatidia with the given number of photoreceptors
SCA3trQ78
SCA3trQ78; Su(z)123/+
SCA3trQ78; Su(z)124/+
one two three four five six seven
f.
wild-type
SCA3trQ78
SCA3trQ78, Su(z)122 / +
SCA3trQ78, Pcl5 / +
SCA3trQ78, E(z)731 / +
g.
Percent of ommatidia with the given number of photoreceptors
SCA3trQ78
SCA3trQ78; Su(z)122/+
SCA3trQ78; Pcl5/+
SCA3trQ78; E(z)731/+
wild-type
one two three four five six seven

Supplementary Figure 4: Retinal degeneration due to pathogenic ATXN3 is suppressed by heterozygous mutations in *Pcl* or *Su(z)12* (Associated with Figure 5)

a, b, c. qPCR analysis demonstrating that heterozygous mutations in (**a**) *Pcl*, (**b**) *Su(z)12,* or (**c**) *E(z)* do not effect transcription of the SCA3trQ78 transgene. (**a**) *Pcl* Mean ± SEM, n=3 biological replicates for mutant, n=5 biological replicates for control. ($F_{3,10}$=0.9, p>0.05, one-way ANOVA). (**b**) *Su(z)12* Mean ± SEM, n=3 biological replicates for mutant, n=5 biological replicates for control. ($F_{3,10}$=0.6, p>0.05, one-way ANOVA). (**c**) *E(z)* Mean ± SEM, n=3 biological replicates for mutant, and control. (p>0.05, t-test).

d. Histogram showing the distribution of photoreceptors (PR) per ommatidium demonstrating mitigation of SCA3trQ78 neuronal loss by the $Pcl^{EY08457}$ and Pcl^{s1859} alleles. Upon SCA3trQ78 expression (red), fewer PR are visible, averaging just 1.72±.06 SEM (n=28 flies) at 21d. In $Pcl^{EY08457}$ heterozygotes (blue) degeneration is mitigated, with the average now 3.08 ± 0.18 SEM (n=19). In Pcl^{s1859} heterozygotes (yellow) degeneration is again mitigated, with the average now 2.87±.18 SEM (n=15 flies, p<.001, Kruskal-Wallis test, Dunn post-test). Genotypes: *rh1-GAL4, UAS-SCA3trQ78/+*. $Pcl^{EY08457}$/+; *rh1-GAL4, UAS-SCA3trQ78/+.* Pcl^{s1859}/+; *rh1-GAL4, UAS-SCA3trQ78/+.*

e. Histogram showing the distribution of PR per ommatidium demonstrating mitigation of SCA3trQ78 neural loss by the *Su(z)12*^{3} *and Su(z)12*^{4} alleles. Upon SCA3trQ78 expression (red), fewer PR are visible, averaging just 1.92±.06 SEM (n=29 flies) at 21d. In *Su(z)12*^{3} heterozygotes (blue), PR cell loss is mitigated to 3.13±.3 SEM (n=18 flies, p<0.01, Kruskal-Wallis test, Dunn post-test). In *Su(z)12*^{4} heterozygotes (yellow), PR cell loss is mitigated to 3.32±.21 SEM (n=18 flies, p<0.01, Kruskal-Wallis test, Dunn post-test). Genotypes: SCA3trQ78 is *rh1-GAL4, UAS-SCA3trQ78/+*. SCA3trQ78; *Su(z)12*^{3}/+ is *rh1-GAL4, UAS-SCA3trQ78 / Su(z)12*^{3}*.* SCA3trQ78; *Su(z)12*^{4}/+ is *rh1-GAL4, UAS-SCA3trQ78 / Su(z)12*^{4}*.*

f. Young animals (3d) heterozygous for PRC2 mutations and expressing SCA3trQ78 have no significant developmental or overproliferation effects of photoreceptors. Each panel shows seven ommatidial units, with only mild photoreceptor cell loss induced by SCA3trQ78. Scale bar indicates 10μm.

g. Histogram showing the distribution of photoreceptors (PR) per ommatidium at 3d in wild-type (black, 7.0 PR), SCA3trQ78 (red, 6.91 ± 0.08 SEM), SCA3trQ78; *Su(z)12*^{2}/+ (blue, 6.92 ± 0.03 SEM), SCA3trQ78; Pcl^{5}/+ (yellow, 6.98 ± 0.03 SEM), SCA3trQ78; *E(z)*^{731}/+ (purple, 6.93 ± 0.07 SEM). n=10 flies for each genotype.

a.

		Genotype/Age		
		wild-type (aged 3d)	wild-type (aged 20d)	*E(z)* mutant (aged 20d)
Temperature	25° (Permissive Temperature)	3 Biological Replicates	3 Biological Replicates	3 Biological Replicates
	29° (Restrictive Temperature)	–	5 Biological Replicates	5 Biological Replicates

b.

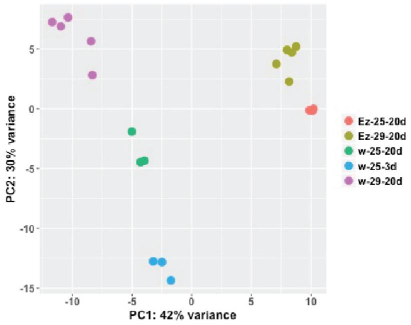

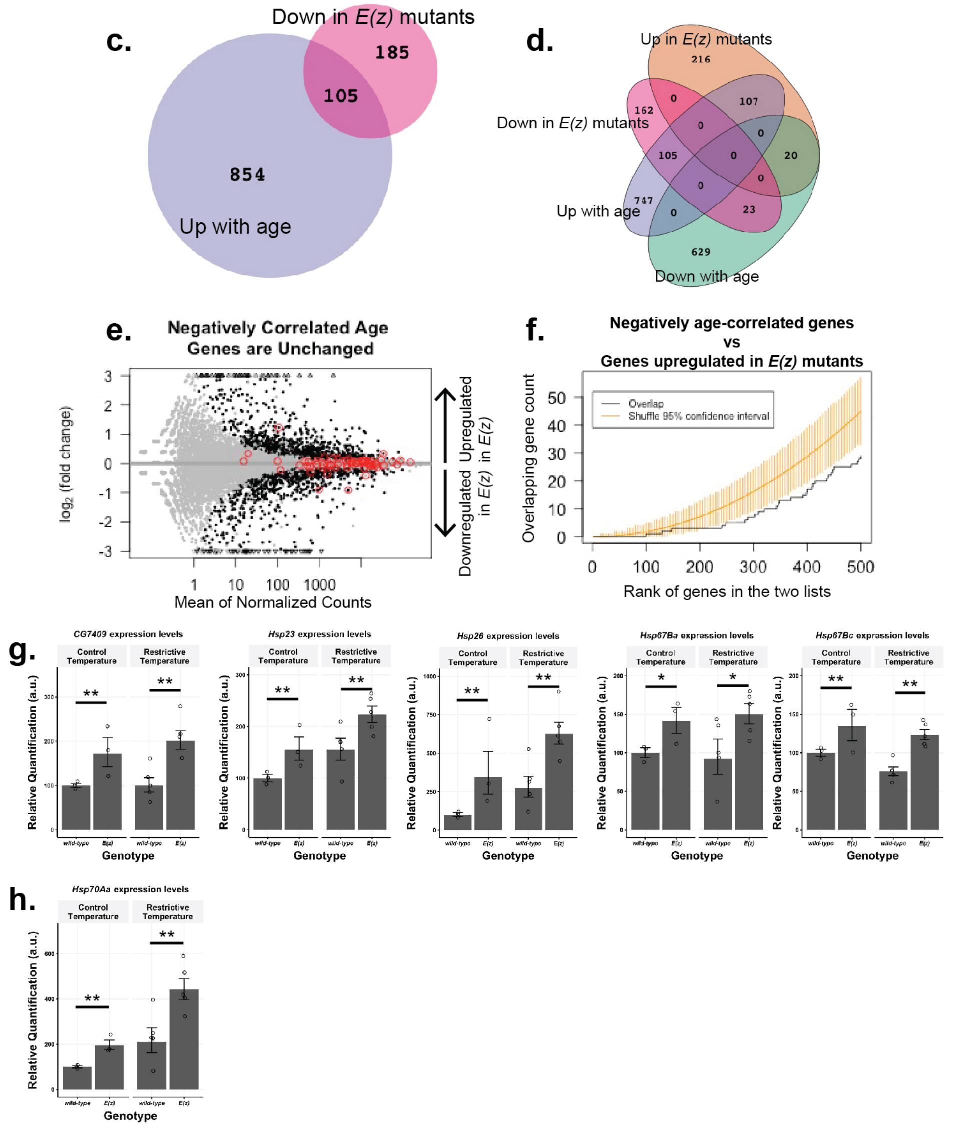

Supplementary Figure 5: RNA-seq quality control and validation (Associated with Figure 7 & 8)

a. Design of RNA-seq experiments. Experimental design and biological replicates. Samples profiled were designed to assess changes in gene expression due to *E(z)* mutation at two separate temperatures to account for a presumptive temperature-sensitive allele, and to assess changes in gene expression due at advanced age.

b. Principal component analysis of RNA-seq samples. Principal component analysis plot on rLog Normalized gene counts using DESeq2. Samples from similar genotype, temperature, and age cluster tightly together.

c. Gene overlaps. Overlap between genes downregulated with age and upregulated in *E(z)*.

d. Gene overlaps between all categories of samples. Overlap between all regulated genes among both age and genotype contrasts.

e. Genes negatively correlated with age show no bias towards upregulation or downregulation in *E(z)* mutants. Grey data points are genes not differentially expressed. Black data points are differentially expressed genes ($p<0.05$). Red circles are genes corresponding to probe sets previously defined as negatively correlated with age[1]. The overlap between genes called either upregulated or downregulated in *E(z)* mutants and the genes negatively-correlated with age is not statistically significant (hypergeometric test).

f. Ordered list analysis of the genes negatively-correlated with age. Two ranked lists of genes were compared: a list of genes ranked by degree of upregulation in *E(z)* mutants (Supplementary Data 1) and the top 500 genes ranked by negative correlation with age[1] (Supplementary Data 2). *E(z)* upregulated genes were ranked by the product of the -log(*p* value) and the moderated $\log_2$ fold-change (high to low), such that genes upregulated in *E(z)* mutants are at the top. All negatively age-correlated genes were ranked according to increasing *p* value of the correlation with age among all genes with a negative correlation, followed by genes with positive correlation which were ranked according to decreasing *p* value. Yellow bars indicate 95% confidence intervals. There was no statistically significant overlap.

g. Small HSPs are upregulated in *E(z)* mutant brains. Regulation of mRNA levels of *CG7409, Hsp23, Hsp26, Hsp67Ba*, and *Hsp67Bc* by *E(z)* was confirmed using qPCR on cDNA prepared from the samples used in RNA-seq. For each gene, the levels are elevated in *E(z)* mutant brains. Asterisks indicate significance of main effect of genotype in two-way ANOVA (**$p<0.01$, *$p<0.05$). *CG7409*: $F_{1,12}=20.9$. *Hsp23*: $F_{1,12}=11.9$. *Hsp26*: $F_{1,12}=18.7$. *Hsp67Ba*: $F_{1,12}=6.1$. *Hsp67Bc*: $F_{1,12}=26.5$.

h. *Hsp70* is upregulated in *E(z)* mutant brains. Regulation of mRNA levels of *Hsp70* by *E(z)* was confirmed using realtime qPCR on cDNA prepared from the samples used in RNA-seq. Asterisks indicate significance of main effect of genotype in two-way ANOVA ($F_{1,12}=14.8$ **$p<.01$). This assay detects three isoforms of *Hsp70* in addition to *Hsp70Ba*, which is upregulated 10-fold in *E(z)* mutants in the RNA-seq data.

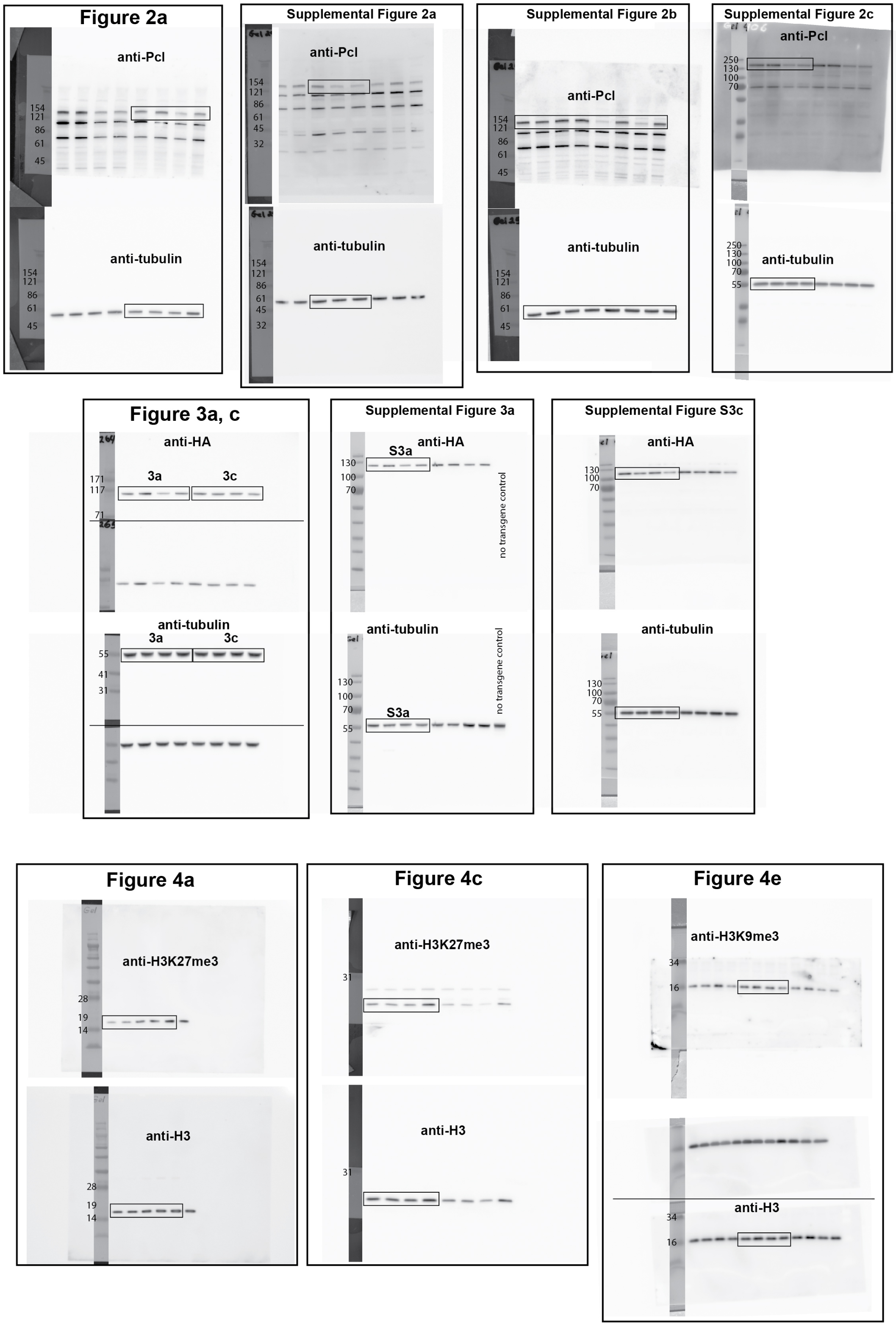

Supplementary Figure 6: Whole Western Blots with size markers
Uncropped Western blot images for each figure. In some cases, the image contains two blots developed simultaneously, in which case the image is bisected by a line. Bands presented in the figure are boxed.

SUPPLEMENTARY TABLES AND LEGENDS

Supplementary Table 1: *Pcl* and *Su(z)12* are predicted targets of *miR-34*
Tabulation of microRNA prediction results from several algorithms. Four prediction algorithms were used to assess whether *miR-34* targets *Pcl* and *Su(z)12* in *Drosophila*, *SUZ12* and *PHF19* in mouse, and *SUZ12, PHF19*, and *MTF2* in humans. "–" indicates that the algorithm does not predict the given gene as a target, and a "+" indicates that it does. Programs used were DIANA [2]; Miranda[3]; Pictar[4]; Targetscan[5]. No target sites were predicted using these programs in *SUZ12* or *MTF2*.

Species	Gene	TargetSCAN	PicTar	miRanda	DIANA-microT
D. melanogaster	*Su(z)12*	-	+	+	+
	Pcl	+	+	+	+
M. musculus	*PHF19*	+	+	-	+
H. sapiens	*PHF19*	+	-	-	+

+ Predicted target
- Not a predicted target

Supplementary Table 2: DAVID analysis of differentially regulated gene sets in brains of *E(z)* and in the brain with age

GO Terms, KEGG Pathways, and InterPro Motifs associated with genes called downregulated in *E(z)* mutant brains, genes upregulated with age, genes upregulated in *E(z)* mutant brains, and genes downregulated with age. The “Category” of each gene set is indicated on the left, “Term” is the name of the given gene set, “Gene Count” is the number of genes called differentially expressed in the given gene set, with the percentage indicated in parentheses. On the right is the Benjamini adjusted *p* value, a threshold for which was set at 0.1.

Genes upregulated in *E(z)* mutants

Category	Term	Gene Count (percent)	Benjamini adjusted p value
InterPro Domains	IPR002068:Alpha crystallin/Hsp20 domain	5 (1.78)	2.62E-02
	IPR020846:Major facilitator superfamily domain	13 (4.63)	3.68E-02
	IPR017972:Cytochrome P450, conserved site	9 (3.2)	4.00E-02
	IPR001128:Cytochrome P450	10 (3.56)	4.18E-02
	IPR001436:Alpha crystallin/Heat shock protein	4 (1.42)	6.14E-02
	IPR002401:Cytochrome P450, E-class, group I	8 (2.85)	7.19E-02
	IPR008978:HSP20-like chaperone	5 (1.78)	7.51E-02
GO: Biological Process	GO:0055114~oxidation-reduction process	23 (8.19)	6.80E-03
	GO:0055085~transmembrane transport	17 (6.05)	3.54E-02
	GO:0046701~insecticide catabolic process	5 (1.78)	9.87E-02
GO: Cellular Component	GO:0031090~organelle membrane	10 (3.56)	1.48E-03
	GO:0005887~integral component of plasma membrane	23 (8.19)	4.82E-03
	GO:0005789~endoplasmic reticulum membrane	12 (4.27)	8.96E-02
GO: Molecular Function	GO:0005506~iron ion binding	14 (4.98)	2.55E-03
	GO:0016705~oxidoreductase activity, acting on paired donors, with incorporation or reduction of molecular oxygen	10 (3.56)	8.17E-03
	GO:0004497~monooxygenase activity	10 (3.56)	9.41E-03
	GO:0016491~oxidoreductase activity	14 (4.98)	1.02E-02
	GO:0020037~heme binding	10 (3.56)	7.49E-02
KEGG Pathways	dme01100:Metabolic pathways	27 (9.61)	5.67E-02
	dme00040:Pentose and glucuronate interconversions	6 (2.14)	6.14E-02

Genes downregulated with Age

Category	Term	Gene Count (percent)	Benjamini adjusted p value
InterPro Domains	IPR005821:Ion transport domain	10 (2.49)	5.51E-03
	IPR006029:Neurotransmitter-gated ion-channel transmembrane domain	7 (1.75)	3.93E-02
	IPR006202:Neurotransmitter-gated ion-channel ligand-binding	7 (1.75)	4.10E-02
	IPR006201:Neurotransmitter-gated ion-channel	7 (1.75)	4.10E-02
	IPR018000:Neurotransmitter-gated ion-channel, conserved site	7 (1.75)	4.37E-02
	IPR001436:Alpha crystallin/Heat shock protein	5 (1.25)	4.40E-02
	IPR004119:Protein of unknown function DUF227	8 (2)	5.15E-02
	IPR015897:CHK kinase-like	8 (2)	5.15E-02
	IPR027359:Voltage-dependent potassium channel, four helix bundle domain	5 (1.25)	7.89E-02
	IPR002068:Alpha crystallin/Hsp20 domain	5 (1.25)	7.89E-02
GO: Biological Process	GO:0015992~proton transport	11 (2.74)	6.63E-06
	GO:0007268~chemical synaptic transmission	16 (3.99)	1.44E-05
	GO:0015986~ATP synthesis coupled proton transport	8 (2)	6.08E-04
	GO:0006813~potassium ion transport	8 (2)	1.02E-02
	GO:0007274~neuromuscular synaptic transmission	12 (2.99)	1.73E-02
	GO:0006811~ion transport	9 (2.24)	2.94E-02
	GO:0006120~mitochondrial electron transport, NADH to ubiquinone	8 (2)	3.38E-02
	GO:0006812~cation transport	8 (2)	3.38E-02
	GO:0048790~maintenance of presynaptic active zone structure	5 (1.25)	3.88E-02
	GO:0009612~response to mechanical stimulus	6 (1.5)	4.05E-02
GO: Cellular Component	GO:0005811~lipid particle	34 (8.48)	5.07E-09
	GO:0030054~cell junction	16 (3.99)	5.41E-06
	GO:0005747~mitochondrial respiratory chain complex I	10 (2.49)	7.64E-04
	GO:0005887~integral component of plasma membrane	38 (9.48)	7.67E-04
	GO:0016021~integral component of membrane	119 (29.68)	2.14E-03
	GO:0005739~mitochondrion	37 (9.23)	2.30E-03
	GO:0005743~mitochondrial inner membrane	14 (3.49)	4.01E-03
	GO:0005886~plasma membrane	47 (11.72)	4.54E-03
	GO:0031430~M band	5 (1.25)	8.01E-03
	GO:0045211~postsynaptic membrane	9 (2.24)	8.38E-03
	GO:0000276~mitochondrial proton-transporting ATP synthase complex, coupling factor F(o)	5 (1.25)	1.05E-02
	GO:0031594~neuromuscular junction	10 (2.49)	1.41E-02
	GO:0000275~mitochondrial proton-transporting ATP synthase complex, catalytic core F(1)	4 (1)	2.21E-02
	GO:0016328~lateral plasma membrane	5 (1.25)	8.85E-02
GO: Molecular Function	GO:0046933~proton-transporting ATP synthase activity, rotational mechanism	9 (2.24)	3.30E-05
	GO:0008553~hydrogen-exporting ATPase activity, phosphorylative mechanism	5 (1.25)	6.47E-02
	GO:0003954~NADH dehydrogenase activity	7 (1.75)	6.81E-02

	GO:0005249~voltage-gated potassium channel activity	6 (1.5)	9.44E-02
KEGG Pathways	dme00190:Oxidative phosphorylation	29 (7.23)	1.25E-12
	dme01100:Metabolic pathways	71 (17.71)	1.46E-10
	dme01230:Biosynthesis of amino acids	12 (2.99)	2.75E-04
	dme01200:Carbon metabolism	15 (3.74)	3.71E-04
	dme04512:ECM-receptor interaction	6 (1.5)	1.19E-03
	dme00220:Arginine biosynthesis	5 (1.25)	9.72E-03
	dme01130:Biosynthesis of antibiotics	17 (4.24)	1.22E-02
	dme00010:Glycolysis / Gluconeogenesis	8 (2)	1.44E-02
	dme00020:Citrate cycle (TCA cycle)	7 (1.75)	1.57E-02
	dme01210:2-Oxocarboxylic acid metabolism	5 (1.25)	1.61E-02
	dme00830:Retinol metabolism	6 (1.5)	1.74E-02
	dme00983:Drug metabolism - other enzymes	7 (1.75)	2.17E-02
	dme00630:Glyoxylate and dicarboxylate metabolism	6 (1.5)	2.24E-02
	dme00053:Ascorbate and aldarate metabolism	5 (1.25)	5.40E-02
	dme00910:Nitrogen metabolism	4 (1)	6.04E-02

Genes Downregulated in *E(z)* Mutants

Category	Term	Gene Count (percent)	Benjamini adjusted *p* value
InterPro Domains	IPR005521:Attacin, C-terminal	5 (1.78)	5.91E-04
	IPR014756:Immunoglobulin E-set	9 (3.2)	0.006035175
	IPR015510:Peptidoglycan recognition protein	5 (1.78)	0.006938317
	IPR002502:N-acetylmuramoyl-L-alanine amidase domain	5 (1.78)	0.007496597
	IPR006619:Peptidoglycan recognition protein family domain, metazoa/bacteria	5 (1.78)	0.007496597
	IPR017331:Peptidoglycan recognition protein, PGRP-S	4 (1.42)	0.008190656
	IPR005520:Attacin, N-terminal	4 (1.42)	0.009995804
GO: Biological Process	GO:0019731~antibacterial humoral response	9 (3.2)	1.30E-06
	GO:0050830~defense response to Gram-positive bacterium	11 (3.91)	5.47E-06
	GO:0007601~visual perception	12 (4.27)	6.64E-06
	GO:0045087~innate immune response	14 (4.98)	8.45E-06
	GO:0009617~response to bacterium	8 (2.85)	6.53E-04
	GO:0009253~peptidoglycan catabolic process	5 (1.78)	0.009387639
	GO:0007602~phototransduction	8 (2.85)	0.012389477
	GO:0016059~deactivation of rhodopsin mediated signaling	5 (1.78)	0.0182863
	GO:0006959~humoral immune response	5 (1.78)	0.021244987
	GO:0042742~defense response to bacterium	7 (2.49)	0.033155188
	GO:0050962~detection of light stimulus involved in sensory perception	3 (1.07)	0.063836943
GO: Cellular Component	GO:0016028~rhabdomere	12 (4.27)	9.60E-09
	GO:0005576~extracellular region	23 (8.19)	0.001892863
	GO:0016027~inaD signaling complex	5 (1.78)	0.00278399
	GO:0005615~extracellular space	20 (7.12)	0.030827809
GO: Molecular Function	GO:0008745~N-acetylmuramoyl-L-alanine amidase activity	5 (1.78)	0.013519092
	GO:0042834~peptidoglycan binding	5 (1.78)	0.025970077
KEGG Pathways:	dme04745:Phototransduction - fly	6 (2.14)	0.001074313

Genes Upregulated with Age

Category	Term	Gene Count (percent)	Benjamini adjusted *p* value
GO: Biological Process	GO:0007052~mitotic spindle organization	11 (2.17)	0.077527726
GO: Cellular Component	GO:0005576~extracellular region	35 (6.90)	4.09E-05
	GO:0005615~extracellular space	32 (6.31)	0.001354075
	GO:0000776~kinetochore	9 (1.78)	0.016590776
	GO:0032133~chromosome passenger complex	4 (0.79)	0.020331765
	GO:0051233~spindle midzone	5 (0.99)	0.069461209
GO: Molecular Function	GO:0005184~neuropeptide hormone activity	12 (2.37)	3.52E-05

Supplementary Table 3: qPCR amplicons
qPCR amplicons listing the target of interest and sequences of the two primers used. Also indicated is the correlation coefficient (R^2) and Efficiency.

Target	Left primer sequence	Right primer sequence	R^2	Efficiency (%)
SCA3trQ78	CAGGACAGAGTTCACATCCATGT	GCCTTACCTAGATCACTCCCAAGT	0.999	104.4
tbp	TAAGCCCCAACTTCTCGATTCC	GCCAAAGAGACCTGATCCCC	0.996	108.3
RpS20	CCGCATCACCCTGACATCC	TGGTGATGCGAAGGGTCTTG	0.999	95.6
pgk	ATCACCAGCAACCAGAGAATTG	TGCCAGGGTGTACTTGATGTT	0.990	103.8
HSP23	TGAGGAGCGCGAAGATGAC	CATAGCGGCGGACAAAGTG	0.995	99.5
HSP67Ba	GTTTCTTGTGTTTTCTTTTCTTGCAT	GTGCTTTTCCCGTCAGCTTT	0.997	97.2
HSP67Bc	CGGCAGCATCATGATCTTGA	CACCCATCCGGGCAATC	0.999	101.5
HSP67Bc	CCACCCGCTGGCAAATC	CGACGACCTGTTTTCCTTTCA	0.999	97.2
CG13133	AGCGCGAGATCGAGATTGA	TCGGACACGCTGCCAAA	0.982	104.9

Supplementary Table 4: Genotyping primers

Genotyping primers indicates primers used to genotype the *E(z)*^{61} and *E(z)*^{731} alleles in a heterozygous background. Column "Allele" indicates the allele being genotyped. Column "L Primer" and "R Primer" indicate the sequence of the primers used in PCR. Column "enzyme" indicates the restriction endonuclease used to resolve the different alleles. Column "+/+ (bp)" indicates the restriction fragment size expected for wild-type DNA amplified with the given primers and digested with the given enzyme. Column "+/- (bp)" indicates the sized expected if the DNA amplified was from a heterozygous animal.

Allele	L Primer	R Primer	enzyme	+/+ (bp)	+/- (bp)
E(z)^{61}	TGCAATACGAAGCAGTGTCCTTGC	CATGAGCAGGTGCTTGTCTGCAAT	*AciI*	148, 70	218, 148, 70
E(z)^{731}	AGCGAGGACTGCGTAAGAAACCTT	GGCAAACCGAATCTTGTTGCCCTT	*DdeI*	310	190, 120

Supplementary Table 5: Plasmid details
Description of construct and primer sequences of cloning methods used to create plasmids used in this work.

Plasmid	Text name	Description	Left Primer Sequence	Right Primer Sequence
**pJRK200**	pMT-Renilla Pcl 3'UTR	Renilla Luciferase reporter for Pcl	CATAggatccACCCTTTGGCATACCGACTCATTATGAATTTTATATTCTTCATTTCCATTATCATTTCTTTTCGTTATTATGTATTAATGAACGCTGCCAACTACGGGCCTCTTTATTTGTTAATGTTTAAAAGTTTATTACCAACCC	AGGTgtcgacTTTTTACAATTTAACATTTTATCGCTTTAATGCTGAACCAATATTTTGAACAATCTTTTTCTATTTggCAgtgGGT TGG TAA TAA ACT TTT AAA CAT TAA CAA ATA AAG AGG CCC
**pJRK202**	pMT-Renilla Su(z)12 3'UTR	Renilla Luciferase reporter for Su(z)12	CATAggatccATC AGT AAT AAT ACA GTG CTT AAC AAG CGG CAG	AGGTgtcgacGTATGTAGTCGTAGGCTCTAGTGATAGTGTTCTTGC
**pJRK204**	pMT-Renilla Pcl 3'UTR sm	Renilla Luciferase reporter for Pcl with seed mutation induced by Quikchange---Quikchange primers listed	gggcctctttatttgttaatgtttaaaagtttattaccaaccatgcatcaaaatagaaaaagattgttcaaaatattggttcagcattaa	ttaatgctgaaccaatattttgaacaatctttttctattttgatgcatggttggtaataaacttttaaacattaacaaataaagaggccc
**pJRK205**	pMT-Renilla Su(z)12 3'UTR sm	Renilla Luciferase reporter for Su(z)12 with seed mutation induced by Quikchange---Quikchange primers listed	gcggagccaaccgcaacaaaagcaataaccattatgcatcagcaaccagtaacaacgc	gcgttgttactggttgctgatgcataatggttattgcttttgttgcggttggctccgc
**pJRK228**	Su(z)12-C-term-HA	Promoter and coding fragment fused to HA (BamHI sited downstream of stop) cloned into pCasper4 vector containing wild-type Su(z)12 3'UTR derived from pJRK202	ttga ggtacc AGCCCATCGTTTGATTGCGTTCAG	accagtggatccTTAAGCGTAATCTGGAACATCGTATGGGTA GTTGCCCACACAGTTGTTGGC AAC
**pJRK235**	Su(z)12-C-term-HA-mutant-3'UTR	Promoter and coding fragment fused to HA (BamHI sited downstream of stop) cloned into pCasper4 vector containing mutated Su(z)12 3'UTR derived from pJRK205	ttga ggtacc AGCCCATCGTTTGATTGCGTTCAG	accagtggatccTTAAGCGTAATCTGGAACATCGTATGGGTA GTTGCCCACACAGTTGTTGGC AAC

Supplementary Table 6: Antibodies & Western parameters

Antibodies and experimental parameters used in western immunoblotting. Pcl and Histones are high pI proteins that required a higher pH transfer buffer. The first few columns list the target, catalog number, Lot, and manufacturer of each antibody used. Column “Pre-Blocking” indicates the blocking buffer used. Column “Gel type” indicates the various gels used for each antigen. Column “Transfer Type / Buffer” indicates the methods used to transfer the proteins to PVDF membrane. Column “Transfer time & temperature” indicates transfer conditions. Column “1° dilution” indicates the concentration of primary antibody used relative to the manufacturer supplied stock. Column “primary antibody buffer” indicates the buffer used in primary antibody incubations. Columns “2° catalog number”, “2° antibody dilution”, and “2° antibody buffer” indicate the parameters used for secondary antibody incubation. After each antibody incubation, the immunoblots were rinsed as indicated in Column “Post-incubation Rinses”

antigen	1° catalog number	Lot	1° manufacturer	Pre-Blocking Buffer	Gel type	Transfer Type / Buffer	Transfer time & temperature	1° dilution	primary antibody buffer	2° catalog number	2° antibody manufacturer	2° antibody dilution	2° antibody buffer	Post-incubation Rinses
Pcl	44520002	G3131-055A01	Novus	1hr in TBST + 5% non-fat dry milk	Laemmli or TGX (Biorad)	Wet / Dunn Carbonate	16-18hr at 4 degrees	1:5000	TBST + 5% non-fat dry milk	111-035-144	Jackson ImmunoResearch	1:5000	TBS + 5% non-fat dry milk	3 times for 5m in TBST
tubulin	#9099S	1	Cell Signalling	1hr in TBS + 5% non-fat dry milk	Various (used for loading control)	Various (used for loading control)	Various (used for loading control)	1:1000	TBS + 5% non-fat dry milk	not applicable	not applicable	not applicable	not applicable	3 times for 5m in TBST
HA	1.2014E+10	11058700	Roche	1hr in PBS + 5% non-fat dry milk	NuPage Bis-Tris (Invitrogen)	Semi-Dry / NuPage Transfer [illegible]	20m at room temperature	1:500	PBS + 5% non-fat dry milk	not applicable	not applicable	not applicable	not applicable	3 times for 5m in TBST
H3K27me3 (Fig 3C,D)	07-449	DAM1662421	Millipore	1hr in TBS + 5% non-fat dry milk	Tris-Tricine	Wet / Dunn Carbonate	16-18hr at 4 degrees	1:30000	TBS	111-035-144	Jackson ImmunoResearch	1:5000	TBS + 5% non-fat dry milk	3 times for 5m in TBST

H3 (Figure 3C,D,E,F)	H3 (Figure 3A,B)	H3K9me3	H3K27me3 (Fig 3A,B)	antigen
#3638S	ab1791	ab8898	07-449	**1° catalog number**
1	852719	GR22415-2	DAM1662421	**Lot**
Cell Signalling	abcam	abcam	Millipore	**1° manufacturer**
1hr in TBS + 5% non-fat dry milk	1hr in TBS + 5% non-fat dry milk	1hr in TBS + 5% non-fat dry milk	1hr in TBS + 5% non-fat dry milk	**Pre-Blocking Buffer**
Tris-Tricine	NuPage Bis-Tris (Invitrogen)	Tris-Tricine	NuPage Bis-Tris (Invitrogen)	**Gel type**
Wet / Dunn Carbonate	Semi-Dry / NuPage Transfer buffer	Wet / Dunn Carbonate	Semi-Dry / NuPage Transfer buffer	**Transfer Type / Buffer**
16-18hr at 4 degrees	20m at room temperature	16-18hr at 4 degrees	20m at room temperature	**Transfer time & temperature**
1:30000	1:5000	1:10000	1:5000	**1° dilution**
TBS + 5% non-fat dry milk	TBS	TBS	TBS	**primary antibody buffer**
115-035-146	111-035-144	111-035-144	111-035-144	**2° catalog number**
Jackson ImmunoResearch	Jackson ImmunoResearch	Jackson ImmunoRes earch	Jackson ImmunoResearch	**2° antibody manufacturer**
1:5000	1:10000	1:10000	1:5000	**2° antibody dilution**
TBS + 5% non-fat dry milk	TBS + 5% non-fat dry milk	TBS + 5% non-fat dry milk	TBS + 5% non-fat dry milk	**2° antibody buffer**
3 times for 5m in TBST	3 times for 5m in TBST	3 times for 5m in TBST	3 times for 5m in TBST	**Post-incubation Rinses**

Supplementary Table 7: Genotypes of flies used in each experiment
Detailed genotypes of animals used in the various figures are given.

Fig.	Text Description	genotype	Stock name	male parent stock	female parent stock	Reference
**2**	*miR-34Δ*	*Df(3R)^{0110}, P{w^{+mC} Fmr1^{+t} miR-277^{+t} =FAFB}4*	Bonini 0110			[1]
**2**	wild-type	*w^{1118}*	FBst0005905			
**3**	*wild-type Su(z)12-C-term HA*	*w^{1118}; P{w^{+mC} Su(z)12$^{+t228T:Ivi\HA}$=Su(z)12 C-termHA}L5 / +*		JKC0744 *w^{1118}*; P{w^{+mC} *Su(z)12$^{+t228T:Ivi\HA}$=Su(z)12 C-termHA}L5 / SM6A*	FBst0005905	
**3**	*miR-34Δ Su(z)12-C-term HA*	*w1118; P{w^{+mC} Su(z)12$^{+t228T:Ivi\HA}$=Su(z)12 C-termHA}L5 / +; Df(3R)^{0110}, P{w^{+mC} Fmr1^{+t} miR-277^{+t} =FAFB}4*		JKC0815 *w1118; P{w^{+mC} Su(z)12$^{+t228T:Ivi\HA}$=Su(z)12 C-termHA}L5 /* SM6A ; *Df(3R)^{0110}, P{w^{+mC} Fmr1^{+t} miR-277^{+t} =FAFB}4*	Bonini 0110 *Df(3R)^{0110}, P{w^{+mC} Fmr1^{+t} miR-277^{+t} =FAFB}4*	
**3**	*wild-type Su(z)12-C-term HA-mutant 3'UTR*	*w$^{1118;}$ P{w^{+mC} Su(z)12$^{+t235T:Ivi\HA}$=Su(z)12 C-termHA NsiI}L3 / +*		JKC0739 *w$^{1118;}$ P{w^{+mC} Su(z)12$^{+t235T:Ivi\HA}$=Su(z)12 C-termHA NsiI}L3 / SM6A*	FBst0005905	
**3**	*miR-34Δ Su(z)12-C-term HA-mutant 3'UTR*	*w$^{1118;}$ P{w^{+mC} Su(z)12$^{+t235T:Ivi\HA}$=Su(z)12 C-termHA NsiI}L3 / + ; Df(3R)^{0110}, P{w^{+mC} Fmr1^{+t} miR-277^{+t} =FAFB}4*		JKC0816 *w$^{1118;}$ P{w^{+mC} Su(z)12$^{+t235T:Ivi\HA}$=Su(z)12 C-termHA NsiI}L3 / SM6A ; Df(3R)^{0110}, P{w^{+mC} Fmr1^{+t} miR-277^{+t} =FAFB}4*	Bonini 0110 *Df(3R)^{0110}, P{w^{+mC} Fmr1^{+t} miR-277^{+t} =FAFB}4*	
**4**	*miR-34Δ*	*Df(3R)^{0110}, P{w^{+mC} Fmr1^{+t} miR-277^{+t} =FAFB}4*	Bonini 0110			[1]
**4**	*wild-type*	*w^{1118}*	FBst0005905			
**5, 6**	*wild-type*	Canton S	FBst0064349			
**5, 6**	*SCA3trQ78*	*rh1-gal4-1, P{w^{+mC} UAS-Hsap\MJD.tr-Q78}c211.2 / +*		FBst0005905	*rh1-gal4-1, P{w^{+mC} UAS-Hsap\MJD.tr-Q78}c211.2 / TM6B,Tb*	
**5, 6**	*SCA3trQ78 Pcl^{5} / +*	Pcl[5], P{neoFRT}42D / +; *rh1-gal4-1, P{w^{+mC} UAS-Hsap\MJD.tr-Q78}c211.2 / +*		FBst0024157	*rh1-gal4-1, P{w^{+mC} UAS-Hsap\MJD.tr-Q78}c211.2 / TM6B,Tb*	
**5, 6**	*SCA3trQ78 Su(z)12^{2} / +*	*rh1-gal4-1, P{w^{+mC} UAS-Hsap\MJD.tr-Q78}c211.2 / Su(z)12^{2}, P{FRT(w^{hs})}2A*		FBst0024159	*rh1-gal4-1, P{w^{+mC} UAS-Hsap\MJD.tr-Q78}c211.2 / TM6B,Tb*	
**5**	*SCA3trQ78 E(z)^{731} / +*	*rh1-gal4-1, P{w^{+mC} UAS-Hsap\MJD.tr-Q78}c211.2 / E(z)^{731}*		JKC0226 *w^{1118}; E(z)^{731} / TM6, Sb*	*rh1-gal4-1, P{w^{+mC} UAS-Hsap\MJD.tr-Q78}c211.2 / TM6B,Tb*	
**7**	*wild-type*	*w^{1118}*	FBst0005905			
**7**	*E(z)*	*w^{1118}; E(z)^{731} / E(z)^{61}*		JKC0226 *w^{1118}; E(z)^{731}/TM6, Sb*	JKC0200 *w^{1118}; E(z)^{61} /TM6, Sb*	
**S2a**	+ / +	*w^{1118}*	FBst0005905			
**S2a**	*Pcl^{4} / Pcl^{5}*	*Pcl^{4} / Pcl^{5}, P{neoFRT}42D*		*Pcl^{4} / CyO*	FBst0024157	Pcl^{4}/CyO gift of Ian Duncan
**S2a**	*Pcl^{4} / Pcl^{1}*	*Pcl^{4} / Pcl^{1}*		*Pcl^{1} / CyO*	*Pcl^{4} / CyO*	Pcl^{1}/CyO gift of Ian Duncan
**S2b**	*wild-type*	*w^{1118}*	FBst0005905			
**S2b**	*daGS-GAL4/+*	*da-GeneSwitch (II) / +*		FBst0005905	*da-GeneSwitch (II)*	daGS-GAL4 gift of Veronica Monnier

**S2b**	daGS-GAL4 / Pcl RNAi$^{TRiP}_{HMS00896}$	da-GeneSwitch (II) / + ; P{TRiP.HMS00896}attP2 / +		FBst0033945	da-GeneSwitch (II)	
**S2b**	daGS-GAL4 / Pcl RNAi$^{TRiP}_{HMS00897}$	da-GeneSwitch (II) / + ; P{TRiP.HMS00897}attP2 / +		FBst0033946	da-GeneSwitch (II)	
**S2c, d**	miR-34Δ	Df(3R)^{0110}, P{w^{+mC} Fmr1^{+t} miR-277^{+t} =FAFB}4	Bonini 0110			[1]
**S2c, d**	wild-type	w^{1118}	FBst0005905			
**S3**	SCA3trQ78	rh1-gal4-1, P{w^{+mC} UAS-Hsap\MJD.tr-Q78}c211.2 / +		FBst0005905	rh1-gal4-1, P{w^{+mC} UAS-Hsap\MJD.tr-Q78}c211.2 / TM6B,Tb	
**S3**	SCA3trQ78 Pcl^{5} / +	Pcl^{5}, P{neoFRT}42D / +; rh1-gal4-1, P{w^{+mC} UAS-Hsap\MJD.tr-Q78}c211.2 / +		FBst0024157	rh1-gal4-1, P{w^{+mC} UAS-Hsap\MJD.tr-Q78}c211.2 / TM6B,Tb	
**S3**	SCA3trQ78 Pcl^{EY08457} / +	P{EPgy2}Pcl^{EY08457} / +; rh1-gal4-1, P{w^{+mC} UAS-Hsap\MJD.tr-Q78}c211.2 / +		FBst0019876	rh1-gal4-1, P{w^{+mC} UAS-Hsap\MJD.tr-Q78}c211.2 / TM6B,Tb	
**S3**	SCA3trQ78 Pcl^{s1859} / +	P{lacW}Pcl^{s1859} / +; rh1-gal4-1, P{w^{+mC} UAS-Hsap\MJD.tr-Q78}c211.2 / +		FBst0012058	rh1-gal4-1, P{w^{+mC} UAS-Hsap\MJD.tr-Q78}c211.2 / TM6B,Tb	
**S3**	SCA3trQ78 Su(z)12^{2} / +	rh1-gal4-1, P{w^{+mC} UAS-Hsap\MJD.tr-Q78}c211.2 / Su(z)12^{2}, P{FRT(w^{hs})}2A		FBst0024159	rh1-gal4-1, P{w^{+mC} UAS-Hsap\MJD.tr-Q78}c211.2 / TM6B,Tb	
**S3**	SCA3trQ78 Su(z)12^{3} / +	rh1-gal4-1, P{w^{+mC} UAS-Hsap\MJD.tr-Q78}c211.2 / Su(z)12^{3}, red^{1}, e*		FBst0005068	rh1-gal4-1, P{w^{+mC} UAS-Hsap\MJD.tr-Q78}c211.2 / TM6B,Tb	
**S3**	SCA3trQ78 Su(z)12^{4} / +	rh1-gal4-1, P{w^{+mC} UAS-Hsap\MJD.tr-Q78}c211.2 / Su(z)12^{4}, P{FRT(w^{hs})}2A, e^{1}		FBst0024469	rh1-gal4-1, P{w^{+mC} UAS-Hsap\MJD.tr-Q78}c211.2 / TM6B,Tb	
**S3**	SCA3trQ78 E(z)^{731} / +	rh1-gal4-1, P{w^{+mC} UAS-Hsap\MJD.tr-Q78}c211.2 / E(z)^{731}		JKC0226 w^{1118}; E(z)^{731}/TM6, Sb	rh1-gal4-1, P{w^{+mC} UAS-Hsap\MJD.tr-Q78}c211.2 / TM6B,Tb	
**S4**	wild-type Su(z)12-C-term HA	w^{1118}; P{w^{+mC} Su(z)12$^{+t228T:Ivi\backslash HA}$=Su(z)12 C-termHA}L3 / +		JKC1458 P{w^{+mC} Su(z)12$^{+t228T:Ivi\backslash HA}$=Su(z)12 C-termHA}L3 / SM6A	FBst0005905	
**S4**	miR-34Δ Su(z)12-C-term HA	w1118; P{w^{+mC} Su(z)12$^{+t228T:Ivi\backslash HA}$=Su(z)12 C-termHA}L3 / +; Df(3R)^{0110}, P{w^{+mC} Fmr1^{+t} miR-277^{+t} =FAFB}4		JKC1438 Su(z)12$^{+t228T:Ivi\backslash HA}$=Su(z)12 C-termHA}L3 / SM6A ; Df(3R)^{0110}, P{w^{+mC} Fmr1^{+t} miR-277^{+t} =FAFB}4	Bonini 0110 Df(3R)^{0110}, P{w^{+mC} Fmr1^{+t} miR-277^{+t} =FAFB}4	
**S4**	wild-type Su(z)12-C-term HA-mutant 3'UTR	w$^{1118;}$ P{w^{+mC} Su(z)12$^{+t235T:Ivi\backslash HA}$=Su(z)12 C-termHA NsiI}L2 / +		JKC1459 w$^{1118;}$ P{w^{+mC} Su(z)12$^{+t235T:Ivi\backslash HA}$=Su(z)12 C-termHA NsiI}L2 / SM6A	FBst0005905	
**S4**	miR-34Δ Su(z)12-C-term HA-mutant 3'UTR	w$^{1118;}$ P{w^{+mC} Su(z)12$^{+t235T:Ivi\backslash HA}$=Su(z)12 C-termHA NsiI}L2 / + ; Df(3R)^{0110}, P{w^{+mC} Fmr1^{+t} miR-277^{+t} =FAFB}4		JKC1437 w[1118]; w$^{1118;}$ P{w^{+mC} Su(z)12$^{+t235T:Ivi\backslash HA}$=Su(z)12 C-termHA NsiI}L2 / SM6A; Df(3R)^{0110}, P{w^{+mC} Fmr1^{+t} miR-277^{+t} =FAFB}4	Bonini 0110 Df(3R)^{0110}, P{w^{+mC} Fmr1^{+t} miR-277^{+t} =FAFB}4	
**S5**	wild-type	w^{1118}	FBst0005905			
**S5**	E(z)	w^{1118}; E(z)^{731} / E(z)^{61}		JKC0226 w^{1118}; E(z)^{731}/TM6, Sb	JKC0200 w^{1118}; E(z)^{61} /TM6, Sb	
