# Supplementary material for: MiR-34 inhibits polycomb repressive complex 2 to modulate chaperone expression and promote healthy brain aging
Source: Nat Commun. 2018 Oct 10;9:4188. doi: 10.1038/s41467-018-06592-5 (PMC6180074; doi:10.1038/s41467-018-06592-5)
Supplement: Supplementary file 2 — Description of Additional Supplementary Files [file 41467_2018_6592_MOESM2_ESM.pdf]

Description of Additional Supplementary Files

File Name: **Supplementary Data 1**
Description: **Lists of genes differentially expressed in *E(z)* mutant brains and in brains with age**

Sheet 1: Genes differentially expressed in *E(z)* brains (EzCalledDE). Lists of differentially expressed genes prepared using the DESeq2 R package, using a Benjamini adjusted *p* value threshold of 0.05 across all lists, with a $\log_2$ fold change threshold of >0.5, for genes that increase in *E(z)* mutant brains, or a $\log_2$ fold change threshold of <-0.5 for genes that decrease in *E(z)* mutant brains. These tables are produced by the results function in the DESeq2 package. "EzCalledDE" lists genes that are either upregulated or downregulated in *E(z)* mutant brains.

Sheet 2: Genes differentially expressed with Age (AgeCalledDE). Lists of differentially expressed genes prepared using the DESeq2 R package, using a Benjamini adjusted *p* value threshold of 0.05 across all lists, with a $\log_2$ fold change threshold of >0.5, for genes that increase with age in brains, or a $\log_2$ fold change threshold of <-0.5 for genes that decrease in the brain with age. These tables are produced by the results function in the DESeq2 package. **"**AgeCalledDE" lists genes that are upregulated or downregulated with age.

For both sheets, column "baseMean" indicates the expression level of the gene. Column "log2FoldChange" indicates the $\log_2$ fold change, with genes upregulated having a positive log2FoldChange, and genes downregulated having a negative log2FoldChange. Column "lfcSE" indicates the standard error associated with the log2FoldChange. Column "stat" indicates the Wald statistic for the given gene. Column "pvalue" indicates the unadjusted *p* value as calculated by the DESEq2 package. Column "Benjamini.correction" indicates the Benjamini adjusted *p* value. Column "ensemble" indicates Ensembl gene name. Column "entrez" indicates Entrez gene name. Column "geneSymbol" indicates abbreviated gene name. Column cg indicates Fly Annotation IDs. Column "Bonf.correction" indicates the Bonferroni adjusted *p* value.

File Name: **Supplementary Data 2**
Description: **Rank list of age-correlated genes.**
List of genes ranked by degree of age-correlation. Genes with a positive correlation with age in the microarray data of Liu *et al.* (2012) were ordered in terms of *p* value, from smallest to largest, followed by genes with a negative correlation with age, which were instead ordered by *p* value from largest to smallest, such that the most negatively-correlated with age genes are at the bottom of the list, while the most positively-correlated with age genes are at the top of the list. Column "Rank" indicates the rank of the gene in the list. Column "ensembl" indicates the gene name. Column "p.value" indicates the significance of the correlation. The first 500 genes in the list, from FBgn0031775 to FBgn0036207, were used in Figure 8c, and show overlap with genes downregulated in *E(z)* mutants. The bottom 500 genes in the list, from FBgn0038929 to FBgn0039900, do not show overlap with genes upregulated in *E(z)* mutants (Supplementary Figure 4f).
